# Structural insights into the substrate-binding proteins Mce1A and Mce4A from *Mycobacterium tuberculosis*

**DOI:** 10.1107/S2052252521006199

**Published:** 2021-07-28

**Authors:** Pooja Asthana, Dhirendra Singh, Jan Skov Pedersen, Mikko J. Hynönen, Ramita Sulu, Abhinandan V. Murthy, Mikko Laitaoja, Janne Jänis, Lee W. Riley, Rajaram Venkatesan

**Affiliations:** a Faculty of Biochemistry and Molecular Medicine, University of Oulu, Oulu, Finland; b Department of Chemistry and Interdisciplinary Nanoscience Center (iNANO), Aarhus University, Aarhus, Denmark; c Department of Chemistry, University of Eastern Finland, Joensuu, Finland; d School of Public Health, University of California, Berkeley, California, USA

**Keywords:** *Mycobacterium tuberculosis*, mammalian cell entry proteins, Mce1, Mce4, substrate-binding proteins, lipids, ABC transporters, crystal structure, SAXS, membrane proteins, protein structure, X-ray crystallography

## Abstract

*Mycobacterium tuberculosis* (*Mtb*), which is responsible for more than a million deaths annually, uses lipids as the source of carbon and energy for its survival in the latent phase of infection. *Mtb* cannot synthesize all of the lipid molecules required for its growth and pathogenicity. Therefore, it relies on transporters such as the mammalian cell entry (Mce) complexes to import lipids from the host across the cell wall. Despite their importance for the survival and pathogenicity of *Mtb*, information on the structural properties of these proteins is not yet available. Each of the four Mce complexes in *Mtb* (Mce1–4) comprises six substrate-binding proteins (SBPs; MceA–F), each of which contains four conserved domains (N-terminal transmembrane, MCE, helical and C-terminal unstructured tail domains). Here, the properties of the various domains of *Mtb* Mce1A and Mce4A, which are involved in the import of mycolic/fatty acids and cholesterol, respectively, are reported. In the crystal structure of the MCE domain of Mce4A (MtMce4A_39–140_) a domain-swapped conformation is observed, whereas solution studies, including small-angle X-ray scattering (SAXS), indicate that all Mce1A and Mce4A domains are predominantly monomeric. Further, structural comparisons show interesting differences from the bacterial homologs MlaD, PqiB and LetB, which form homohexamers when assembled as functional transporter complexes. These data, and the fact that there are six SBPs in each *Mtb mce* operon, suggest that the MceA–F SBPs from Mce1–4 may form heterohexamers. Also, interestingly, the purification and SAXS analysis showed that the helical domains interact with the detergent micelle, suggesting that when assembled the helical domains of MceA–F may form a hydrophobic pore for lipid transport, as observed in EcPqiB. Overall, these data highlight the unique structural properties of the *Mtb* Mce SBPs.

## Abstract 2

The mammalian cell entry genes (*mceA*–*mceF*) encode the substrate-binding proteins of the lipid-transporting Mce complexes in mycobacteria. The MCE domain of Mce4A has been crystallized as a domain-swapped dimer with the signature β-barrel fold. Solution studies show that the domains of Mce1A and Mce4A are predominantly monomeric and suggest that the helical domain is involved in lipid interactions.

## 1. Introduction   

**Figure 1:**
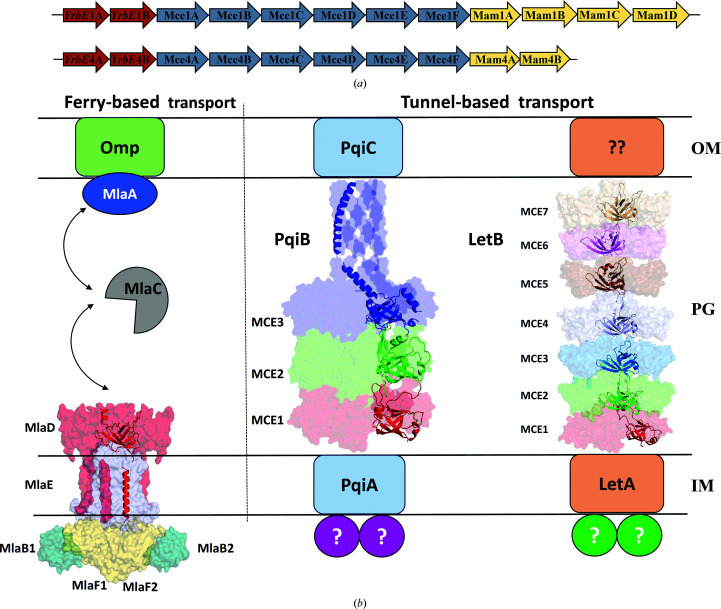
(*a*) Part of the *mce1* and *mce4* operons of *Mtb* encoding permeases (YrbEA–B), SBPs (MceA–F) and Mam proteins. (*b*) Lipid-transporter complexes from *E. coli* [EcMlaFEDB (PDB entry 6zy2), EcPqiB (PDB entry 5uvn) and EcLetB (PDB entry 6v0c)] for which structural information has been reported (Ekiert *et al.*, 2017 ▸; Isom *et al.*, 2020 ▸; Tang *et al.*, 2021 ▸). Lipid transport by the EcMlaFEDB complex depends on a ferry-based lipid-transport mechanism, whereas the EcPqiB and EcLetB complexes facilitate a tunnel-based transport mechanism (Ekiert *et al.*, 2017 ▸; Kamischke *et al.*, 2019 ▸; Coudray *et al.*, 2020 ▸; Isom *et al.*, 2020 ▸; Liu *et al.*, 2020 ▸; Mann *et al.*, 2020 ▸). OM is the outer membrane of *Mtb*, PG is peptidoglycan and IM is the inner membrane. In each of these transporters the MCE domains are assembled into hexameric rings that stabilize the assembled homohexameric complexes.

*Mycobacterium tuberculosis* (*Mtb*) is a deadly intracellular pathogen that causes the disease tuberculosis (Tb), which is responsible for more than a million deaths every year. Approximately one quarter of the population of the world is latently infected with *Mtb* (World Health Organization, 2018 ▸). *Mtb* can persist in a host for months to years. It is one of the very few bacteria which rely on host lipids as the source of energy and carbon for intracellular survival. Additionally, it also converts these lipid molecules into precursors for cell-membrane remodeling, cell-wall homeostasis and ultimately pathogenesis (Cantrell *et al.*, 2013 ▸; Santangelo *et al.*, 2016 ▸; Queiroz & Riley, 2017 ▸; Zhang *et al.*, 2018 ▸; Fenn *et al.*, 2020 ▸; Alonso *et al.*, 2020 ▸). This property might be most relevant during the intra-phagosomal latent stage of infection (Pandey & Sassetti, 2008 ▸). The mammalian cell entry (Mce) proteins encoded by the *mce1*, *mce2*, *mce3* and *mce4* operons [Fig. 1 ▸(*a*)] are important proteins that play a pivotal role in the import of lipid molecules and *Mtb* pathogenesis (Cole *et al.*, 1998 ▸). These operons are comprised of 10–14 genes each. Their name is based on the initial observation that a DNA fragment (corresponding to Mce1A) from *Mtb* (strain H37Ra), when expressed in *Escherichia coli*, caused cell entry of *E. coli* into HeLa cells (Arruda *et al.*, 1993 ▸). Similar to Mce1A, the expression of Mce3A and Mce4A in *E. coli* also provides *E. coli* with the ability to invade HeLa cells (El-Shazly *et al.*, 2007 ▸; Saini *et al.*, 2008 ▸). Nevertheless, subsequent research has shown that the primary role of these proteins concerns lipid transport, and in addition Mce proteins are also involved in modulating host cell signaling, cell-wall homeo­stasis and cell-membrane remodeling (Alonso *et al.*, 2020 ▸; Fenn *et al.*, 2020 ▸; Queiroz & Riley, 2017 ▸; Santangelo *et al.*, 2016 ▸) and are therefore important for the survival and pathogenesis of *Mtb*.

In terms of lipid transport in *Mtb*, Mce proteins are characterized as ABC transporters. It is now well demonstrated that Mce1 is involved in the transport of mycolic acid/fatty acids and Mce4 imports cholesterol. *Mtb* that is disrupted in the Mce2 operon accumulates sulfolipid-1 at levels nearly ten times that of wild-type *Mtb* during stationary growth (Pandey & Sassetti, 2008 ▸; Casali & Riley, 2007 ▸; Marjanovic *et al.*, 2011 ▸). The substrate specificity of the Mce3 complex is still unknown. These studies suggested that the *mce* operons encode the permeases (YrbEA and YrbEB) and the substrate-binding proteins (SBPs) for the formation of the ABC transporter (Casali & Riley, 2007 ▸; Perkowski *et al.*, 2016 ▸). In addition, the *mce1*, *mce3* and *mce4* operons code for Mce-associated membrane proteins (Mam, also known as Mas), which probably stabilize the Mce complexes (Perkowski *et al.*, 2016 ▸). The ATPase of this ABC transporter is proposed to be encoded by the *mceG* gene (also known as *mkl*), which is located elsewhere in the genome (Joshi *et al.*, 2006 ▸).

Although important functions of Mce proteins from *Mtb* have been established, no detailed protein-level characterization and structural information are available on these proteins from *Mtb* or any other actinobacterial species. This is mainly due to difficulties in the recombinant expression and purification of these membrane proteins. Homologs of the Mce SBPs from *E. coli* (EcMlaD, EcPqiB and EcLetB) and *Acinetobacter baumannii* (AbMlaD) have recently been characterized (Ekiert *et al.*, 2017 ▸; Kamischke *et al.*, 2019 ▸; Coudray *et al.*, 2020 ▸; Isom *et al.*, 2020 ▸; Liu *et al.*, 2020 ▸; Mann *et al.*, 2020 ▸) [Fig. 1 ▸(*b*)]. A common feature of each of these proteins is that they all contain a conserved domain of approximately 100 residues, now referred to as the MCE domain, which is characterized by a seven-stranded β-barrel fold, although the sequence identity of these domains is very low. EcMlaD and AbMlaD have a single MCE domain, which forms a homohexamer in the assembled complex. EcPqiB and EcLetB have three and seven MCE domains, respectively, in a single polypeptide, which form stacks of homohexamers in the assembled complex. In contrast, each of the four *Mtb mce* operons encodes six different Mce SBPs and these SBPs have more domains compared with the *E. coli* and *A. baumannii* homologs. In this study, our main objectives have been to identify the various domains of MtMce1A and MtMce4A, guided by sequence analysis and secondary-structure prediction, and to perform a detailed structural characterization. The results of these studies show that the SBPs of *Mtb* have unique structural properties that differ from those of their bacterial counterparts.

## 2. Materials and methods   

### 2.1. Biochemicals   

The genomic DNA of *Mtb* H37Rv was purchased from ATCC. Phusion DNA polymerase and the restriction enzymes used for cloning were purchased from Thermo Scientific (Massachusetts, USA) and New England Biolabs. The Ni–NTA chromatography resin was obtained from Qiagen (Hilden, Germany).

### 2.2. Cloning, expression and purification of MtMce1A–1F and MtMce4A–4F   

Individual MtMce1A–1F and MtMce4A–4F genes were PCR-amplified using *Mtb* H37Rv genomic DNA as the template with specific primers (Supplementary Tables S1 and S2). Each amplicon was cloned into pETM11 vector (EMBL) using a restriction-based cloning method, resulting in an N-terminal His_6_ tag followed by a TEV protease site, the MceA–F gene and a C-terminal His_6_ tag. For protein expression, the plasmid was transformed into *E. coli* BL21-RIPL competent cells. Overnight cultures were grown at 30°C until the OD_600_ reached 0.6, and expression of the protein was induced with 0.5 m*M* isopropyl β-d-1-thiogalactopyranoside (IPTG) at 16°C overnight. The cells were harvested by centrifugation at 4000*g*. The bacterial pellet was resupended in the desired lysis buffer with a suitable detergent (Supplementary Table S3). The cells were lysed by sonication and the lysate was centrifuged at 15 000*g* and 4°C for 30 min. The supernatant was then filtered (0.45 µm; Millipore) and the proteins were allowed to bind to the Ni^2+^–NTA matrix for 1 h. The beads were washed, and bound proteins were eluted from the Ni–NTA column using 400 m*M* imidazole in the elution buffer (Supplementary Table S3). At this step, the concentration of the detergent was reduced to 5 m*M*. The eluted protein was analyzed by 12% or 18% SDS–PAGE, concentrated (spin concentrator, molecular-mass cutoff 30 kDa; Millipore) and injected onto a size-exclusion chromatograpy (SEC) column (Superdex 200 10/300 or Superdex 75 HiLoad 16/600; GE Healthcare).

### 2.3. Expression and purification of the MtMce1A and MtMce4A domains   

Based on the secondary-structure analysis, MtMce1A and MtMce4A domain constructs were generated. They were cloned in pETM11 using restriction-free cloning methods: the constructs were named according to the secondary-structural features: (i) MCE domain (MtMce1A_36–148_ and MtMce4A_39–140_), (ii) MCE+Helical+Tail domain (MtMce1A_38–454_ and MtMce4A_36–400_), (iii) Helical+Tail domain (MtMce1A_126–454_ and MtMce4A_121–400_), (iv) MCE+Helical domain (MtMce1A_38–325_ and MtMce4A_39–320_) and (v) Tail domain (MtMce4A_321–400_). The expression and purification protocols were similar to those used for the corresponding full-length proteins. Only the MCE-domain constructs (MtMce1A_36–148_ and MtMce4A_39–140_) are soluble in the absence of detergents, and different buffers were used for lysis and elution (Supplementary Table S3) when purifying these domains.

For selenomethionine (SeMet)-labeled MtMce4A_39–140_, the construct was transformed into an auxotrophic strain of *E. coli* (B834), which was grown according to the protocol from Molecular Dimensions (Ramakrishnan *et al.*, 1993 ▸). The expression and purification protocols were similar to those for native MtMce4A_39–140_. SeMet incorporation was confirmed by electrospray ionization liquid chromatography–mass spectrometry (ESI LC-MS), which showed 100% incorporation of SeMet into the protein.

### 2.4. SEC-MALS of the MtMce1A and MtMce4A domains   

SEC-MALS analysis of the purified MtMce1A and MtMce4A domains was carried out using a SEC column coupled to a miniDAWN TREOS light-scattering system (Wyatt Technologies). Purified protein at approximately 5–6 mg ml^−1^ was loaded onto a pre-equilibrated Superdex 200 10/300 column using an autosampler at a rate of 0.4 ml min^−1^ at 4°C in a Shimadzu HPLC/FPLC system. The samples were then passed through a refractive-index (RI) detector, a UV detector and subsequently through the MALS detector. The cumulative data collected from the UV, MALS and RI detectors were analyzed using the *ASTRA* software (Wyatt Technologies). The protein-conjugate analysis method was used to analyse the proteins that were complexed with detergent. The detergent was considered as a modifier and the recommended d*n*/d*c* value of 0.1473 ml g^−1^ for *n*-dodecyl β-d-maltoside (DDM) was used for the protein-conjugate analysis. Analysis of the soluble MtMce1A_36–148_ and MtMce4A_39–140_ constructs was performed without using the protein-conjugate protocol.

To understand the effect of heat and higher ionic strength on the oligomeric state of the MCE domain, purified MtMce4A_39–140_ was subjected to buffer exchange [0.1 *M* 2-(*N*-morpholino)ethanesulfonic acid (MES), 0.7 *M* ammonium sulfate pH 6.0] using a 10 kDa molecular-mass cutoff Amicon concentrator. MtMce4A_39–140_ was heated to 50°C in a thermocycler, with an initial 1 min incubation at 20°C followed by a 0.8°C increase per minute up to 50°C and a final incubation at 50°C for 1 min. The heated protein was then centrifuged at 10 000*g* for 5 min and the supernatant was injected onto a Superdex 200 10/300 column pre-equilibrated with a buffer consisting of 0.1 *M* MES, 0.7 *M* ammonium sulfate pH 6.0. The column was coupled to a MALS detector and was analyzed further to obtain the molecular mass.

### 2.5. Circular-dichroism (CD) spectroscopy of the MtMce1A and MtMce4A domains   

The MtMce1A and MtMce4A domains were diluted in water to obtain a lower buffer and salt concentration. The protein concentration used for CD measurements (Chirascan CD spectrophotometer, Applied Photophysics, Surrey, UK) was 0.05 mg ml^−1^. Secondary-structure calculations of the CD spectra of the MtMce1A and MtMce4A domains purified with DDM and without DDM were performed using the *CDNN* and *BestSel* software packages, respectively (Micsonai *et al.*, 2015 ▸, 2018 ▸). For the determination of the thermal melting temperature (*T*_m_), the sample was heated from 22 to 92°C at a rate of 1°C min^−1^. The melting curves were calculated by comparing the spectra from 190 to 280 nm with the global fit analysis protocol as implemented in the *Global*3 software from Applied Photophysics.

### 2.6. Native mass spectrometry of the MtMce1A_36–148_ and MtMce4A_39–140_ domains   

MtMce1A_36–148_ and MtMce4A_39–140_ were buffer-exchanged into 20 m*M* ammonium acetate pH 6.8 using PD Miditrap G-25 columns (GE Healthcare, Sweden). Mass spectra were measured on a 12 T Bruker solariX XR FT-ICR mass spectrometer using an Apollo-II electrospray ion source (Bruker Daltonics, Bremen, Germany). The instrument was calibrated using sodium perfluoroheptanoic acid (NaPFHA) clusters and was operated with the *FTMS Control* 2.2 software. The mass spectra were further analyzed using the *DataAnalysis* 5.1 software.

### 2.7. SAXS analysis of MtMce1A and MtMce4A domains   

#### 2.7.1. Data collection   

SAXS data for the purified Mce1A and Mce4A domains were collected on the B21 beamline at Diamond Light Source (DLS), UK. Data were collected based on the standard protocols for inline SEC-SAXS and batch-mode measurement using a PILATUS 2M two-dimensional detector at a sample-to-detector distance of 4.014 m and a wavelength of 0.99 Å. Inline SEC-SAXS measurements were collected for domains purified in the presence of the detergent DDM (MtMce1A_38–325_, MtMce1A_126–454_, MtMce1A_38–454_, MtMce4A_39–320_, MtMce4A_121–400_ and MtMce4A_36–400_) at an initial concentration of 5 mg ml^−1^ as SEC can separate the protein–detergent complexes and the empty micelles (Berthaud *et al.*, 2012 ▸). Batch-mode measurements were collected for MtMce1A_38–148_ and MtMce4A_39–140_ at 2 and 1 mg ml^−1^, respectively, with bovine serum albumin (BSA) as a control. For each batch-mode concentration, 25 frames were collected.

#### 2.7.2. Data processing   

Data processing and analysis was performed using the *ScÅtter* and *ATSAS* software packages (Franke *et al.*, 2017 ▸). The 2D data were averaged to give a 1D data set of intensity, *I*(*q*), versus *q*, where *q* is the modulus of the scattering vector. The scattering of the buffer was subtracted from the protein scattering using *ScÅtter*. The data were rebinned using in-house-developed software (Vilstrup *et al.*, 2020 ▸) to be approximately equidistantly spaced on a logarithmic *q* scale. The radius of gyration (*R*_g_), forward scattering *I*(0) and maximum particle distance (*D*_max_) were calculated using *PRIMUS*. The molecular weight was calculated based on two methods: volume of correlation (Rambo & Tainer, 2013 ▸) and *SAXSMoW* (Piiadov *et al.*, 2019 ▸; Supplementary Tables S4, S5 and S6). *Ab initio* shape was generated using *DAMMIN* (Svergun, 1999 ▸). For MtMce4A_39–140_, the compact monomer was generated from residues 32–106 of chain *A* and residues 107–145 of chain *B* of the crystal structure. The elongated monomer corresponds to chain *B* of the crystal structure. These were further provided as a template in *Robetta* to add the missing residues (Raman *et al.*, 2009 ▸; Song *et al.*, 2013 ▸). For MtMce1A_36–148_, the entire compact and elongated models were generated with *Robetta* using the MtMce4A_39–140_ compact and elongated crystal structures as the template. The models were evaluated against the experimental data using an in-house-written program (Steiner *et al.*, 2018 ▸; Vilstrup *et al.*, 2020 ▸). The helical and tail domains of MtMce1A_38–325_, MtMce1A_126–454_, MtMce1A_38–454_, MtMce4A_39–320_, MtMce4A_121–400_ and MtMce4A_36–400_ were generated using *I-TASSER*. Summaries of the data-collection and analysis parameters are provided in Supplementary Tables S4, S5 and S6.

#### 2.7.3. Detergent and protein model fitting for MtMce1A_38–325_, MtMce1A_126–454_, MtMce1A_38–454_, MtMce4A_39–320_, MtMce4A_121–400_ and MtMce4A_36–400_   

The SAXS data for the complexes of DDM with the various constructs were also analyzed using in-house-developed software. The program is based on the methods described previously (Kaspersen *et al.*, 2014 ▸; Steiner *et al.*, 2018 ▸; Vilstrup *et al.*, 2020 ▸; Calcutta *et al.*, 2012 ▸). The DDM micellar structure is represented by Monte Carlo points in a triaxial core-shell structure with super-ellipsoidal shape with shape parameter *t* = 3 (Maric *et al.*, 2017 ▸), and the protein is represented by the atoms in the PDB structures. When the protein overlaps with the core-shell structure, the corresponding Monte Carlo points were removed. The volume of the core was estimated from the number of points and the point density, and the aggregation number was calculated by dividing the core volume by the volume of a C_12_ chain (353 Å^3^). The shell contains both DDM headgroups and solvating buffer, and the thickness of the shell was fixed at 10 Å. In practice, the aggregation number was kept fixed and the lengths of the long axis and of one of the short axes were optimized, whereas the length of the third axis was calculated from these two and the aggregation number. The Monte Carlo points were assigned an excess scattering length corresponding to the electron densities of C_12_ tails and heads for points in the core and in the shell, respectively, taking into account the glycerol content of the buffer. Similarly, the excess scattering length of the atoms of the protein was adjusted taking the glycerol into account. The scattering of a hydration layer was added to the protein in the places where it is not in contact with the micelle. The protein structure was divided into three domains, namely the MCE, helical and tail domains, to allow rigid-body refinement. The domains (MCE+Helical, Helical+Tail and MCE+Helical+Tail, respectively, for the three constructs) were connected by soft restraints as described in Vilstrup *et al.* (2020 ▸). The algorithm for generating the micelle, including estimates of the excess scattering length, were checked by fitting a data frame from pure micelle from the elution profile, and gave a satisfactory fit.

The SAXS data for all constructs have a deep minimum around *q* = 0.1 Å^−1^ followed by a pronounced secondary maximum. This behavior is qualitatively very similar to that of pure DDM micelles, and the first tests revealed that such a *q* dependence could not be obtained when the protein penetrates significantly into the core of the micelles. Further tests showed that reasonable agreement with the SAXS data was obtained when the helix of the protein was along the long axis of the DDM micelle. Therefore, starting structures with this position were used in the optimizations. Additionally, a soft restraint that keeps the helix in contact with the micelle was introduced. The structure was optimized by random searches, initially with large amplitudes, which were gradually decreased during optimization (Vilstrup *et al.*, 2020 ▸). For each structure ten independent runs were performed, each with 4000 cycles of optimization. The structure with the best agreement with the SAXS data in terms of reduced χ^2^ was selected as the resulting structure. Initially the aggregation numbers were estimated from the SEC-MALS results, however, in some cases this did not give good fits to the SAXS data. Therefore, the aggregation number was varied in a reasonable range for these cases.

### 2.8. Crystallization, data collection, structure determination and structure refinement of MtMce4A_39–140_   

Purified MtMce4A_39–140_ and SeMet-labeled MtMce4A_39–140_ were concentrated to 7.5 mg ml^−1^ in protein buffer (Table 1 ▸) and used in all of the crystallization experiments. Crystallization was performed using the sitting-drop vapor-diffusion method at three different drop ratios (100:150, 150:150 and 150:100 nl protein:reservoir solution) at 22°C. Crystals were observed in all three drop ratios when using 100 m*M* sodium HEPES, 100 m*M* LiCl_2_, 20% PEG 400 pH 7.5 as the reservoir solution for native MtMce4A_39–140_ and using 100 m*M* MES, 700 m*M* ammonium sulfate pH 6.0 as the reservoir solution for SeMet-labeled MtMce4A_39–140_. The native MtMce4A_39–140_ and SeMet-labeled MtMce4A_39–140_ crystals were transferred to reservoir solution supplemented with 20% ethylene glycol and 25% glycerol, respectively, for a few minutes and the crystals were subsequently flash-cooled in liquid nitrogen.

The data for both the native MtMce4A_39–140_ and SeMet-MtMce4A_39–140_ crystals were collected on the BioMAX beamline at MAX IV, Lund, Sweden at 2.9 and 3.6 Å resolution, respectively (Table 1 ▸). Data processing and scaling were performed using *XDS* (Kabsch, 2010 ▸) and *AIMLESS* (Evans & Murshudov, 2013 ▸), respectively, which suggested that the space group was *P*6_1_ or *P*6_5_. The SeMet-labeled MtMce4A_39–140_ structure was solved by SeMet SAD phasing using the *CRANK*2 (Skubák & Pannu, 2013 ▸) pipeline with 20 selenium sites. Subsequently, space group *P*6_5_ was chosen based on its better figure of merit. The model obtained from the *CRANK*2 pipeline was completed iteratively by model building using *Coot* (Emsley *et al.*, 2010 ▸) and refinement calculations using *Phenix* (Liebschner *et al.*, 2019 ▸), resulting in a model with an *R*_work_ and *R*_free_ of 0.34 and 0.37, respectively. This model consisted of two swapped dimers in the asymmetric unit. This model was subsequently used as the search model for expert-mode molecular-replacement calculations (Expert-MR) in *Phaser* (McCoy *et al.*, 2007 ▸) to determine the structure of native MtMce4A_39–140_. The obtained molecular-replacement model was then used as an initial model for autobuilding in *Phenix* (Liebschner *et al.*, 2019 ▸). The structure was further refined iteratively using several cycles of manual model building in *Coot* and refinement in *Phenix*. The final refinement steps gave an *R*_work_ and *R*_free_ of 0.19 and 0.23, respectively. This model of native MtMce4A_39–140_ was then again used to refine the SeMet-labeled MtMce4A_39–140_ structure, giving a final *R*_work_ and *R*_free_ of 0.21 and 0.24, respectively (Table 1 ▸).

## 3. Results and discussion   

### 3.1. *Mtb* MceA–F SBPs have a conserved four-domain architecture   

**Figure 2:**
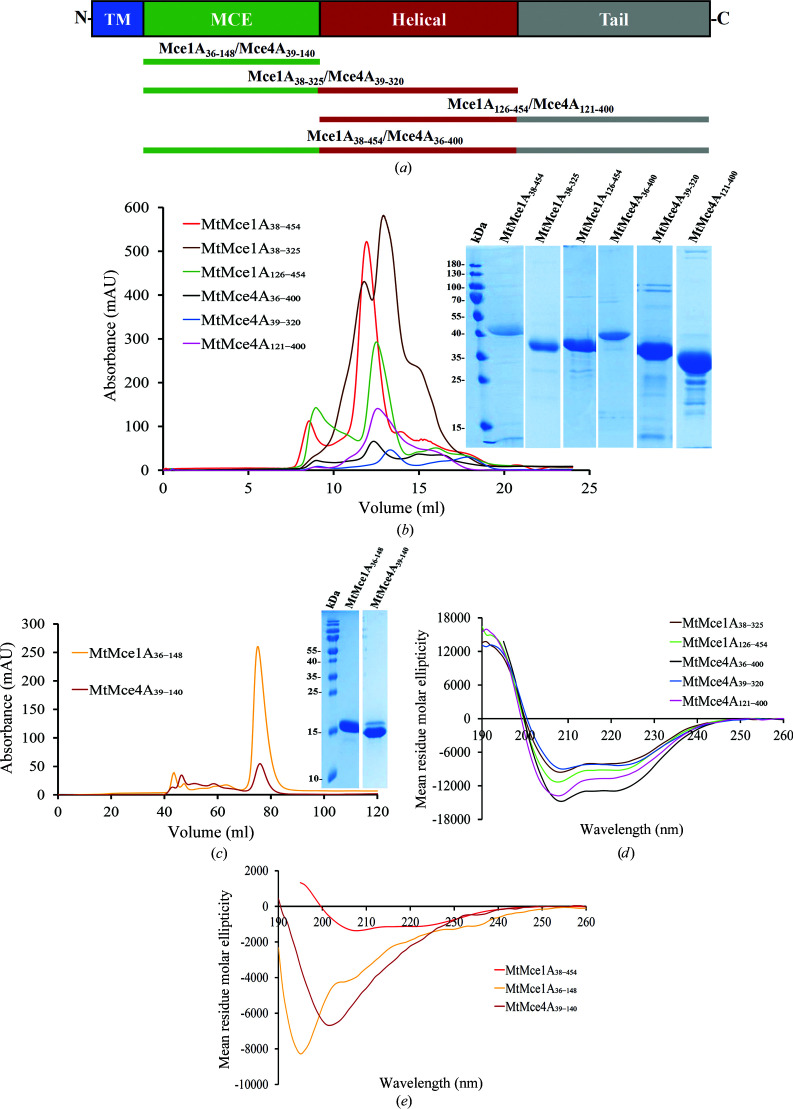
(*a*) The domains of the Mce SBPs. The Mce SBPs are characterized by having four domains referred to as the transmembrane (TM), MCE, helical and tail domains. The constructs of MtMce1A and MtMce4A used in this study are shown below in the same color coding. (*b*) Size-exclusion chromatography (SEC) elution profiles of MtMce1A_38–325_, MtMce1A_126–454_ and MtMce1A_38–454_ and of MtMce4A_39–320_, MtMce4A_121–400_ and MtMce4A_36–400_ on a 24 ml Superdex 200 10/300 column. The protein samples were analyzed by 12% SDS–PAGE (inset). (*c*) SEC elution profiles of MtMce1A_36–148_ and MtMce4A_39–140_ on a 120 ml Superdex 75 HiLoad 16/600 column. The protein samples were analyzed by 18% SDS–PAGE (inset). (*d*) CD spectra of MtMce1A_38–325_ (brown), MtMce1A_126–454_ (green), MtMce4A_36–400_ (black), MtMce4A_39–320_ (blue) and MtMce4A_121–400_ (pink). (*e*) CD spectra of MtMce1A_38–454_ (red), MtMce1A_36–148_ (yellow) and MtMce4A_39–140_ (maroon).

A comparative sequence analysis and secondary-structure prediction (Supplementary Figs. S1 and S2) of the MceA–F SBPs from *Mtb* Mce1–4 suggest that despite their very low sequence identity (∼20% or less; Supplementary Tables S7 and S8) they have a conserved domain architecture, such that each of them has four domains [Fig. 2 ▸(*a*)]. The first domain is an N-terminal transmembrane (TM) domain (∼30–40 amino acids), which is predicted to form a single transmembrane helix, followed by a second domain with ∼100 amino acids mainly composed of β-strands (seven in total), referred to as the MCE domain. The third domain is predicted to mainly consist of long helices (∼200 amino acids) and this domain is therefore referred to here as the helical domain. The fourth domain is predicted to be an unstructured domain and is referred to as the tail domain. Interestingly, the length of the tail domain varies between six and 260 amino acids between the various MtMceA–F SBPs, while the order and length of the other domains is well conserved. Additionally, the tail domains of MtMce1C, MtMce1D, MtMce4D and MtMce4F are proline-rich. Moreover, MtMce1E, MtMce2E, MtMce3E and MtMce4E contain a conserved sequence motif (referred to as the lipobox) in their N-terminus (Sutcliffe & Harrington, 2004 ▸).

Recently, the Mce SBP homologs MlaD from *E. coli* and *A. baumannii* and PqiB and LetB from *E. coli* have been characterized [Fig. 1 ▸(*b*)] (Ekiert *et al.*, 2017 ▸; Kamischke *et al.*, 2019 ▸; Coudray *et al.*, 2020 ▸; Isom *et al.*, 2020 ▸; Liu *et al.*, 2020 ▸; Mann *et al.*, 2020 ▸; Tang *et al.*, 2021 ▸). These homologs vary amongst themselves and also are different when compared with the *Mtb* Mce SBPs in terms of their length and the architecture of the domains (Fig. 1 ▸). For example, the length of the helical domain varies from ∼15 residues in EcLetB to ∼43 in EcMlaD and ∼134 in EcPqiB. Comparatively, the helical domain of the *Mtb* Mce SBPs is much longer than those of any of the *E. coli* homologs. Also, the *Mtb* Mce SBPs and EcMlaD have only a single MCE domain in the polypeptide chain, whereas EcPqiB and EcLetB have three and seven MCE domains, respectively, in a single polypeptide (Ekiert *et al.*, 2017 ▸; Fig. 1 ▸). In addition, the unstructured tail domain of the mycobacterial Mce SBPs is not present in any of the *E. coli* homologs. The role of this tail domain is not understood.

### 3.2. The MCE domain is the only soluble domain of MtMce1A and MtMce4A   

All six of the SBPs encoded in the Mce1 and Mce4 operons (MtMce1A–1F and MtMce4A–4F) were recombinantly expressed in *E. coli* and purified (Supplementary Section S1 and Supplementary Fig. S3) in the presence of detergents. Given that all of these SBPs are predicted to have a similar domain architecture and secondary structure, further detailed domain-level characterization was performed for MtMce1A and MtMce4A. From secondary-structure predictions, the domain constructs of MtMce1A and MtMce4A categorized as MCE (MtMce1A_36–148_ and MtMce4A_39–140_), MCE+Helical (MtMce1A_38–325_ and MtMce4A_39–320_), Helical+Tail (MtMce1A_126–454_ and MtMce4A_121–400_) and MCE+Helical+Tail (MtMce1A_38–454_ and MtMce4A_36–400_) domains were successfully expressed in *E. coli* and screened to evaluate their solubility in the presence and absence of detergents.

Interestingly, the MCE domains of both MtMce1A and MtMce4A (MtMce1A_36–148_ and MtMce4A_39–140_) were the only soluble constructs in the absence of detergents. The MCE+Helical+Tail and MCE+Helical as well as Helical+Tail constructs of MtMce1A and MtMce4A could only be purified in the presence of detergent, even though the transmembrane domain had been deleted in all of these constructs [Figs. 2 ▸(*b*) and 2 ▸(*c*)]. Additionally, extension of the soluble MCE constructs with one (MtMce4A_39–154_) or two (MtMce4A_39–190_) helical domains resulted in insolubility, indicating that the helical domain requires detergent for its solubility. This could be because under physiological conditions the helical domain is either embedded in the hydrophobic region of the cell wall or might be involved in interactions with the lipid substrates. The CD curves of the MtMce1A and MtMce4A domains [Figs. 2 ▸(*d*) and 2 ▸(*e*)] indicated mixtures of α-helical and β-sheet content for all of the MtMce1A and MtMce4A domains purified with DDM, whereas the soluble constructs (MtMce1A_36–148_ and MtMce4A_39–140_) showed a typical β-sheet-dominated spectrum (Supplementary Table S9).

### 3.3. MtMce4A_39–140_ crystallizes as a domain-swapped dimer   

**Figure 3:**
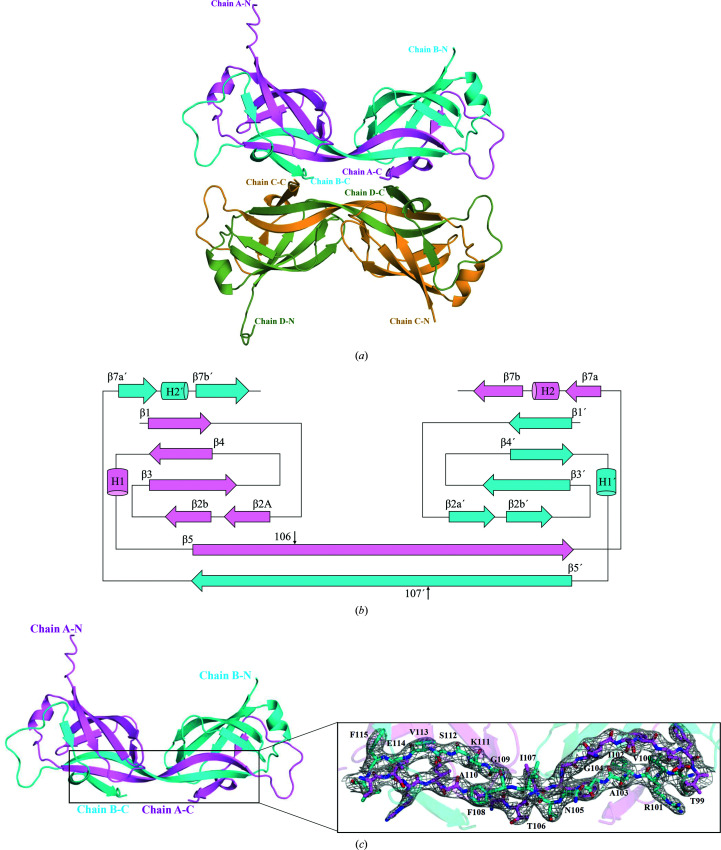
(*a*) Crystal structure of MtMce4A_39–140_ with four molecules in the asymmetric unit. (*b*) Topology of the MtMce4A_39–140_ domain-swapped dimer. β-­Strands are shown as arrows and helices as cylinders. The secondary structures of chain *A* and chain *B* are shown in pink and cyan, respectively. The secondary-structure elements and residue numbers for chain *B* are indicated with primes. The residues after the black vertical arrow are involved in domain swapping. (*c*) The domain-swapped dimer residues of β5 and β5’ are highlighted and shown in the inset. The 2*F*_o_ − *F*_c_ electron-density map contoured at 1.5σ is shown as a gray mesh. These residues are important for the arrangement of the domain-swapped dimer.

Structural studies were initiated on MtMce1A_38–454_ and MtMce4A_36–400_ as well as the soluble MCE domains MtMce1A_38–148_ and MtMce4A_39–140_. Despite extensive trials, only MtMce4A_39–140_ crystallized readily in several conditions in space group *P*6_5_. Given the low sequence identity of MtMce4A_39–140_ to homologous proteins (∼15%), the structure of MtMce4A_39–140_ was determined using SeMet SAD phasing. The data-collection and data-processing statistics are reported in Table 1 ▸. Although Matthews coefficient calculations suggest the presence of 6–8 molecules in the asymmetric unit, assuming a solvent content of about 50%, the solved structure showed that only four molecules are present in the asymmetric unit, corresponding to a solvent content of about 71%. The structure was refined at 2.9 Å resolution (Table 1 ▸). Interestingly, further refinement and model building of the structure revealed that the four molecules of the asymmetric unit are formed by two domain-swapped dimers [Fig. 3 ▸(*a*)]. The electron-density map clearly defines the loops in the regions that define the swapping of the C-terminal part [Fig. 3 ▸(*c*)]. The domain-swapped dimer is formed by the extension of residues 107–141 from one molecule into the other molecule. The swapped region contains two β-strands and an extended loop [Fig. 3 ▸(*a*)].

The secondary structure mainly consists of antiparallel β-strands, forming a β-barrel-like structure. The topology diagram for the swapped dimer is shown in Fig. 3 ▸(*b*). The residues involved in formation of the seven-stranded β-barrel are Thr40–Ser46 (β1), Leu52–Met54 (β2a), Lys59–Gly65 (β2b), Ile65–Ser74 (β3), Arg81–Asp87 (β4), Thr99–Thr106 (β5), Ile107–Ile116 (β5′; considered as the sixth β-strand), His131–Val132 (β7a′) and Val137–Glu141 (β7b′). The residues from 107 to 141 are exchanged between the two monomers to complete the signature MCE fold. The overall structure has visible electron density for all of the residues corresponding to MtMce4A_39–140_ except for the N-terminal residues 1–31 and C-terminal residues 143–146. The latter residues correspond to residues encoded by the vector region.

### 3.4. MtMce1A and MtMce4A are predominantly monomeric in solution   

Given that the MCE domain of prokaryotes exists as a homohexamer in all of the recent studies, the domain-swapped dimer of MtMce4A_39–140_ was surprising (Ekiert *et al.*, 2017 ▸; Kamischke *et al.*, 2019 ▸; Coudray *et al.*, 2020 ▸; Isom *et al.*, 2020 ▸; Liu *et al.*, 2020 ▸; Mann *et al.*, 2020 ▸; Tang *et al.*, 2021 ▸). Therefore, this raised the question as to whether or not the domain-swapped dimer of MtMce4A_39–140_ is physiologically relevant. In order to verify the oligomeric state of MtMce4A_39–140_ in solution, SEC multi-angle light scattering (SEC-MALS) studies were conducted. Interestingly, all of the purified MtMce1A and MtMce4A domains were predominantly monomeric in nature (Table 2 ▸ and Supplementary Figs. S4 and S5). The MtMce1A and MtMce4A domains purified in DDM showed two peaks in the elution profile corresponding to the protein–detergent complex (PDC) and empty detergent micelles, whereas MtMce1A_36–148_ and MtMce4A_39–140_, which are soluble and were purified without DDM, have a single scattering peak corresponding to the monomeric molecular mass (Supplementary Figs. S4 and S5). As the SEC-MALS analysis showed that both MtMce1A_36–148_ and MtMce4A_39–140_ are monomeric in solution and MtMce4A_39–140_ is a domain-swapped dimer in the crystal structure, the oligomeric states of MtMce1A_36–148_ and MtMce4A_39–140_ were also determined by native mass spectrometry (MS) at two different concentrations (5 and 50 µ*M*). These studies further confirmed that both MtMce1A_36–148_ and MtMce4A_39–140_ are monomeric in solution at both concentrations (Supplementary Figs. S6 and S7).

Interestingly, comparison of the secondary-structure content of MtMce4A_39–140_ calculated from the CD spectrum with the crystal structure showed a higher β-sheet content (39%) in the crystal than from the CD spectra in solution (28%; Supplementary Table S10), indicating that the protein has more secondary structure in the crystallized condition. Moreover, thermal melting analysis of MtMce1A_36–148_ and MtMce4A_39–140_ showed that they undergo heat-induced conformational changes (Supplementary Section S1 and Supplementary Figs. S8 and S9).

Since the domain-swapped dimer is only observed in the crystallization condition, the purified MtMce4A_39–140_ was exchanged into crystallization buffer and analyzed by SEC-MALS. Surprisingly, SEC-MALS analysis also showed only the presence of monomeric MtMce4A_39–140_ in the crystallization buffer (Supplementary Fig. S10). However, dimer formation was observed when MtMce4A_39–140_ was heated slowly to 50°C in the crystallization buffer (0.7 *M* ammonium sulfate; Supplementary Fig. S10). These observations suggest that incubation of this protein solution with the crystallization solution at 22°C probably facilitated the protein in attaining a different conformation, including the formation of a domain-swapped dimer. The dimer appears to be selectively crystallized, for example favored by better crystal contacts, compared with the monomer. There are other examples of full-length proteins and truncated domains which exist in different oligomeric states in solution but occur as domain-swapped dimers in the crystalline phase. These examples include barnase, cyanovirin-N, the N-terminal domain of Spo0A and the SH3 domain of Eps8, to name a few (Yang *et al.*, 1999 ▸; Lewis *et al.*, 2000 ▸; Radha Kishan *et al.*, 1997 ▸).

### 3.5. Elongated conformation of MtMce1A_36–148_ and MtMce4A_39–140_ in solution   

**Figure 4:**
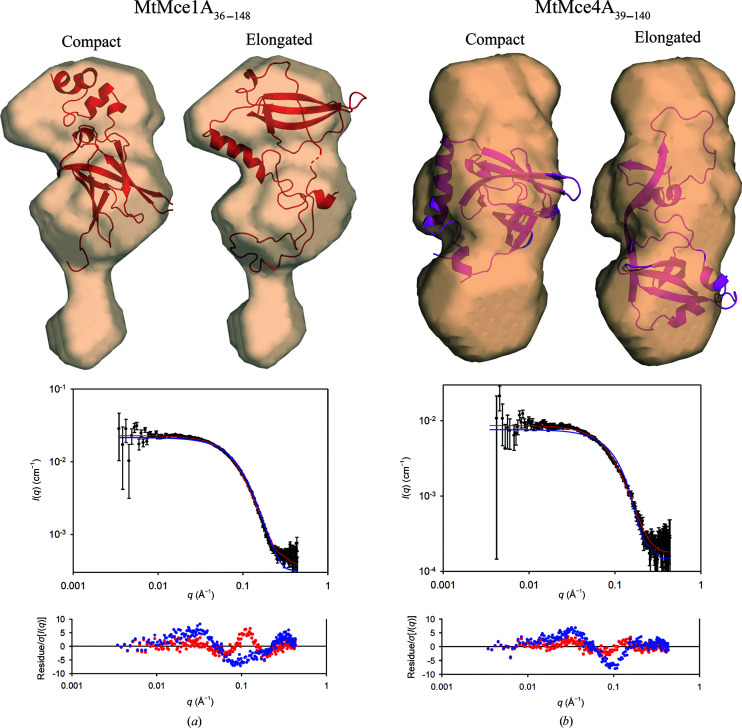
(*a*) The *ab initio* shape generated by *DAMMIN* for MtMce1A_36–148_ superposed on the compact (left) and elongated (right) monomeric models of MtMce1A_36–148_. The corresponding fits of the experimental SAXS data (black) to the elongated (red) and compact (blue) monomers are shown below. (*b*) The *ab initio* shape generated by *DAMMIN* for MtMce4A_39–140_ superposed on the compact (left) and elongated (right) monomeric models of MtMce4A_39–140_. The corresponding fits of the experimental SAXS data (black) to the elongated (red) and compact (blue) monomers are shown below.

The domain-swapped dimer is only observed in the crystals. Therefore, to understand the structures of MtMce1A_36–148_ and MtMce4A_39–140_ in solution, SAXS experiments were performed. The measured intensities *I*(*q*) are displayed as a function of the modulus, *q*, of the scattering vector. Structural parameters calculated from the scattering intensities are given in Supplementary Table S4. The radius of gyration (*R*_g_) and maximum interatomic distances (*D*_max_) were determined to be 21.6 and 70 Å for MtMce1A_36–148_ and 21.7 and 80 Å for MtMce4A_39–140_, respectively [Supplementary Figs. S13(*c*) and 13(*d*)]. Interestingly, the determined *D*_max_ for both MtMce1A_36–148_ and MtMce4A_39–140_ is much higher than the maximum diameter of monomeric EcMlaD (35 Å), pointing towards an elongated structure for both of the proteins. Further, the *ab initio* molecular shapes reconstructed by *DAMMIN* indicate that both MtMce1A_36–148_ and MtMce4A_39–140_ attain an elongated shape under the purified conditions [Figs. 4 ▸(*a*) and 4 ▸(*b*)]. From SEC-MALS and SAXS, we know that the proteins exist as monomers in solution. Therefore, the *ab initio* shape of MtMce4A_39–140_ was fitted with two types of MtMce4A_39–140_ monomer: a compact monomer consisting of residues 32–106 from chain *A* and 107–145 from chain *B* of the crystal structure, and an elongated monomer consisting only of chain *A* as observed in the crystal structure. The missing N-terminal tag and linker sequences were modeled in these molecules using *Robetta*, as explained in Section 2. The χ^2^ values of the compact and elongated models calculated against the experimental SAXS data were 10.0 and 2.0, respectively [Fig. 4 ▸(*b*)]. Similarly, in the case of MtMce1A_36–148_, a template-based model (obtained from *Robetta*) was used to fit the SAXS data, and the χ^2^ values for the compact and elongated models were 14.0 and 11.0, respectively [Fig. 4 ▸(*a*)], here also slightly favoring the elongated model. Further, the domain-swapped region of MtMce1A_36–148_ was optimized by rigid-body refinement and this improved the χ^2^ to 4.2. In summary, the elongated models fit relatively better than the compact model in both cases (Fig. 4 ▸). Taken together, these SAXS studies suggest that both MtMce1A_36–148_ and MtMce4A_39–140_ are in an elongated conformation in solution under the purified conditions, and the presented elongated models derived from the crystal structure in Fig. 4 ▸ represent one of the possible elongated conformations in solution. Nevertheless, in the crystals the MCE fold is still conserved despite its domain-swapped dimer conformation.

### 3.6. Comparison of the MtMce4A_39–140_ structure with the *E. coli* and *A. baumannii* homologs   

Recently, structures of homologs of Mce SBPs from *E. coli* (EcMlaD, EcPqiB and EcLetB) and *A. baumannii* (AbMlaD) have been determined (Isom *et al.*, 2020 ▸; Tang *et al.*, 2021 ▸; Ekiert *et al.*, 2017 ▸; Coudray *et al.*, 2020 ▸; Kamischke *et al.*, 2019 ▸) [Fig. 1 ▸(*b*)]. Based on these homohexameric structures, two different mechanisms of lipid transport have been reported. The first is the Mla complex ferry transport mechanism, in which the Mla operon carries a single Mce gene (MlaD) with a single MCE domain. In this case, the lipids are shuttled between MalaFEDB and MlaA–OmpF by a shuttle protein (MlaC; Ekiert *et al.*, 2017 ▸). The second is the LetB and PqiB tunnel transport mechanism, in which LetB forms a long stack of seven homohexameric MCE domains one above the other connecting the inner and outer membranes, with a central channel mediating lipid transport (Ekiert *et al.*, 2017 ▸; Isom *et al.*, 2020 ▸; Liu *et al.*, 2020 ▸). Like LetB, PqiB also forms a central pore that is formed by three stacked Mce homohexamers, with their long C-terminal helix forming a narrow channel for lipid transport.

In comparison to the homologs from *E. coli* (EcMlaD, EcPqiB and EcLetB) and *A. baumannii* MlaD (AbMlaD) (Ekiert *et al.*, 2017 ▸; Coudray *et al.*, 2020 ▸; Isom *et al.*, 2020 ▸; Kamischke *et al.*, 2019 ▸; Liu *et al.*, 2020 ▸; Mann *et al.*, 2020 ▸), the overall MCE fold with a seven-stranded β-barrel is conserved in the domain-swapped dimer of MtMce4A_39–140_. Notably, part of the MCE fold in MtMce4A_39–140_ is completed by domain swapping. Therefore, we used the compact monomer for structural analysis and comparison. The compact monomer is formed by residues 32–106 of chain *A* and residues 107–145 of chain *B*, whereas the model of the elongated monomer is formed by residues 32–145 of chain *A*.

**Figure 5:**
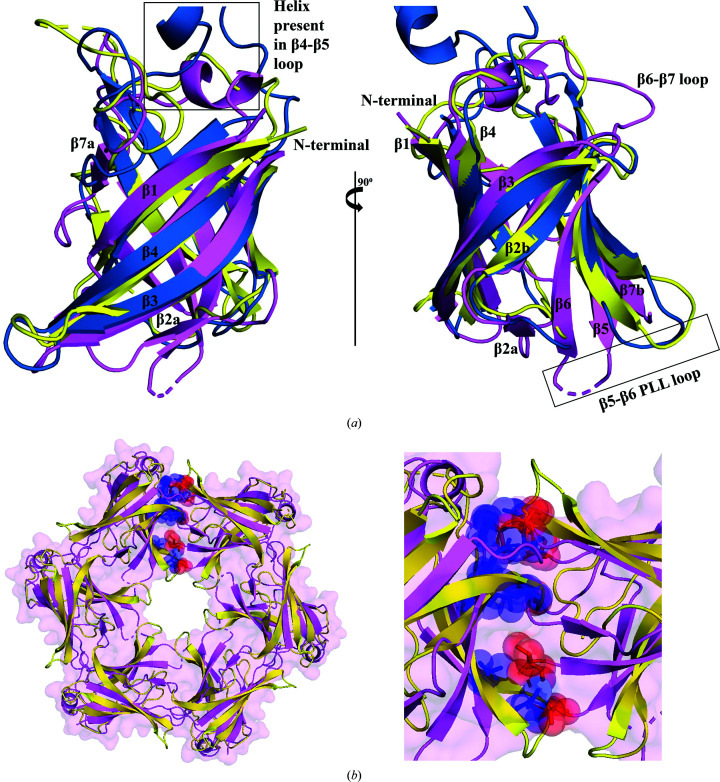
(*a*) Structural superposition of MCE domains from *Mtb* (MtMce4A_39–140_; pink), *E. coli* (EcMlaD; PDB entry 5uw2; yellow) and *A. baumannii* (AbMlaD; PDB entry 6ic4; blue). (*b*) Cartoon representation of the hypothetical homohexamer of MtMce4A_39–140_ (pink) generated based on the EcMlaD homohexamer (yellow). The residues from two monomers (chains *A* and *B*) involved in the steric clashes are shown as spheres and sticks in blue and red. These clashes are between β2–β3 loop residues Lys61, Tyr62 and Arg63 of chain *A* and β3–β4 loop residues Ser76, Gyl77 and Gln79 of chain *B*, between β5 strand residue Ala103 of chain *A* and β5–β6 loop residue Ile107 of chain *B*, between β6 strand residue Glu114 of chain *A* and β5–β6 loop residue Ala50 of chain *B*, and between β7 strand residue Leu140 of chain *A* and β5–β6 loop residues Thr106 and Ile107 of chain *B*.

Superposition of the C^α^ atoms of the MCE domain of MtMce4A on EcMlaD and AbMlaD yields root-mean square-deviations (r.m.s.d.s) of 1.7 and 2.6 Å, respectively [Fig. 5 ▸(*a*)]. The sequence identity between the MtMCE domain and the *E. coli* and *A. baumanni* MCE Mla domains is lower than 15% (Supplementary Fig. S11). The overall topology of the protein is conserved, with conformational differences mainly in the loop regions and a few other secondary-structural elements. For example, β2a (52–54) is only present in MtMce4A_39–140_ and not in EcMlaD and AbMlaD. The β4–β5 loop has an extra helix in MtMce4A_39–140_ and AbMlaD (a 45-residue insertion) and this helix is absent in EcMlaD. The β6–β7 loop in MtMce4A_39–140_ is a proline-rich loop, whereas it is lined with charged residues in EcMlaD and AbMlaD. In addition, density for the β6–β7 loop is missing in the EcMlaD crystal structure and is present in MtMce4A_39–140_ and AbMlaD. β7a and β7b are connected by a helix in MtMce4A_39–140_ and by a loop in EcMlaD, while β7a is absent in AbMlaD. The homologous β7b strand is much smaller in EcMlaD and AbMlaD compared with MtMce4A_39–140_.

Similarly, MtMce4A_39–140_ was superposed with the MCE domains of EcPqiB1–3 and EcLetB1–7 monomers (Supplementary Fig. S11). The sequence identity between the MtMCE domains and the EcPqiB1–3 MCE domains ranges from 7% to 18% and that between the MtMCE domains and the EcLetB1–7 domains ranges from 13 to 26% (Supplementary Fig. S11). The superposition showed that the β-barrel fold is conserved and the observed differences are mainly in the loop regions. For example, β2a (52–54) is unique to MtMce4A_39–140_ and is absent throughout in EcPqiB1–3 and EcLetB1–7. The β3–β4 loop conformation present on the exterior surface varies amongst MtMce4A_39–140,_ EcPqiB1–3 and EcLetB1–7. It is notable that the length of β3–β4 loop remains constant (four residues) in all of the MCE domains except EcPqiB3, which has 18 residues in the loop. Furthermore, the β6–β7 loop in MtMce4A_39–140_ has a different conformation compared with EcPqiB1–3 and EcLetB1–7. Amongst the available Mce SBP structures and MceA–F from MtMce1–4, MtMce4A_39–140_ has a maximum number of proline residues in the β6–β7 loop. The role of this proline-rich loop is not understood.

The β5 strand and the hydrophobic β5–β6 loop (also referred to as the pore-lining loop; PLL) involved in forming the hydrophobic central pore have a different conformation in MtMce4A_39–140_, which contrasts with EcMlaD and AbMlaD. The PLL (β5–β6 loop) comprising the hydrophobic channel is much longer (16–27 residues) in EcPqiB1–2 and EcLetB1–7 when compared with MtMce4A_39–140_ (five residues). We found that the PLL in EcPqiB3 has only seven residues and it is the only MCE domain in EcPqiB and EcLetB which shares this feature with MtMce4A_39–140_. Interestingly, the conformation of the PLL varies throughout the MCE domains of EcPqiB1–3 and EcLetB1–7 (Supplementary Fig. S12). The central pore of all of the reported Mce SBP hexamers is comprised of highly hydrophobic residues, also known as the PLL, which allows the transport of small hydrophobic lipid molecules across the membranes. The variation in the length of the PLL depends on the transport mechanism followed by the particular Mce complex as well as the number of MCE domains that are present. For example, EcMlaD and AbMlaD have a smaller PLL (six residues) and they have a single MCE domain and follow a ferry-based transport mechanism. In comparison, the PLL is longer in EcPqiB and EcLetB (17–27 residues), which have three and seven MCE domains and follow a tunnel-based lipid-transport mechanism. It has been reported that the PLL of EcPqiB1–3 and EcLeTB1–7 follows the pattern φ*xx*φφ, where φ denotes a hydrophobic amino acid and *x* represents any amino acid (Isom *et al.*, 2020 ▸). Although this pattern is followed in MtMce1A (_112_ATTVF_116_), it does not align with the other Mce SBPs from *Mtb* (Supplementary Fig. S11). Instead, the other *Mtb* Mce SBPs follow the pattern *xxx*φφ in the PLL, which aligns with EcMlaD and AbMlaD. Notably, the *Mtb* Mce SBPs and the MlaDs have only one MCE domain. Nevertheless, this conserved ‘duo’ of consecutive hydrophobic residues in the MtMce1A–F and MtMce4A–F SBPs indicate the formation of a hydrophobic pore. In addition, the helical domain of the MtMceA–F SBPs also has a high number of hydrophobic residues, although a clear ‘motif’ is not observed. The monomeric nature of MtMce4A_39–140_ is in contrast to the other Mce proteins (MlaD, PqiB and LetB) from *E. coli* and *A. baumannii*, which form a homohexamer (Ekiert *et al.*, 2017 ▸; Kamischke *et al.*, 2019 ▸; Coudray *et al.*, 2020 ▸; Isom *et al.*, 2020 ▸; Liu *et al.*, 2020 ▸; Mann *et al.*, 2020 ▸; Tang *et al.*, 2021 ▸). Based on EcMlaD, we modeled a hypothetical homohexamer of MtMce4A_39–140_ (Fig. 5 ▸ *b*) by superposing the MtMce4A_39–140_ monomer onto each of the six EcMlaD monomers. Interestingly, the protein–protein interface of the modeled homohexamer of MtMce4A_39–140_ has multiple steric clashes which will preclude the formation of homohexamers in MtMce4A [the clashes between chains *A* and *B* are shown in Fig. 5 ▸(*b*)]. These clashes are absent in EcMlaD, AbMlaD, EcPqiB1–3 and EcLetB1–7, where homohexamers are formed. Overall, these comparisons show the different properties of MtMce4A_39–140_, although the core MCE fold is well conserved.

### 3.7. The helical domains of MtMce1A and MtMce4A interact with the DDM core   

As shown by our purification and SEC-MALS studies, only the MCE domain is soluble without the use of detergent; all other Mce1A and Mce4A domains require detergents, although none of these domain constructs contained the transmembrane domains. Therefore, to further understand the interaction of detergents with these domains, SAXS measurements were performed for the longer MtMce1A and MtMce4A domain constructs in SEC-inline mode (Supplementary Tables S5 and S6). The elution profile has two peaks: one for the PDCs and one for the empty micelles, showing that the PDCs and empty micelles are separated during SEC. The SAXS scattering data of the PDCs display a minimum at a scattering-vector modulus of 0.1 Å^−1^ followed by a broad bump. This suggests that the protein is interacting with the nearly intact detergent micelle. However, the scattering from the PDCs is distinctly different from that of empty micelles due to the additional strong scattering from the protein in the PDCs. This makes the forward scattering much greater for the PDCs (Supplementary Fig. S14). The empty micelle has a deeper minimum compared with the minima of PDCs around *q* = 0.1 Å^−1^. Additionally, the detergent is differently organized in the PDCs and therefore the shape of the secondary maximum also differs for PDCs and empty micelles (Supplementary Fig. S14; Kaspersen *et al.*, 2017 ▸; Pedersen *et al.*, 2020 ▸). The scattering contribution of the detergent micelle interferes with the protein scattering, and detergent scattering cannot be separated from protein scattering in PDCs. Therefore, instead of calculating *ab initio* shapes for the protein, both the detergent micelle and the protein parts were modeled and the SAXS data for the MtMce1A and MtMce4A complex constructs with DDM were also modeled using in-house-developed software (Vilstrup *et al.*, 2020 ▸).

**Figure 6:**
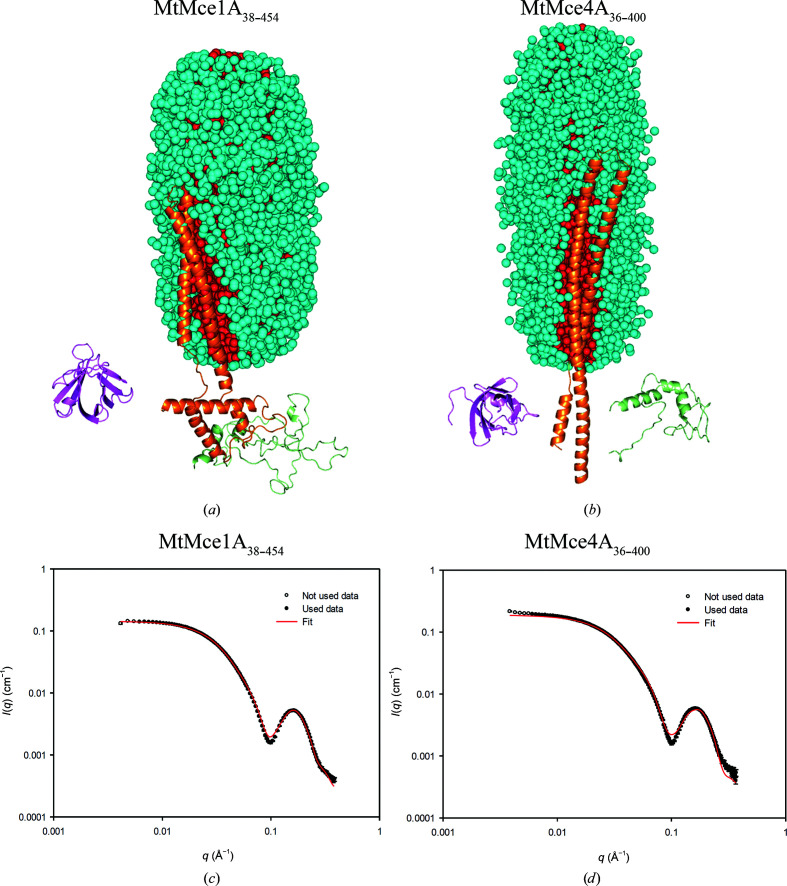
The coiled-coil models of (*a*) MtMce1A_38–454_ and (*b*) MtMce4A_36–400_ interacting with the core of the DDM micelle. The DDM molecules are represented as spheres and the protein is represented as a cartoon. The model is represented by three domains: the MCE domain, helical domain and tail domain. The MCE domains of MtMce1A_38–454_ and MtMce4A_36–400_ are shown in magenta. The helical and tail domains are represented in orange and green, respectively, in both models. The DDM core and shell are shown in red and cyan, respectively. The MCE and tail domains are not fully connected to the helical domain, as we used soft restraints between the three domains while fitting the model to the SAXS data to allow some flexibility. (*c*, *d*) The fits (red line) of the experimental SAXS data (black dots) to the proposed models of (*c*) MtMce1A_38–454_ and (*d*) MtMce4A_36–400_ are shown.

**Figure 7:**
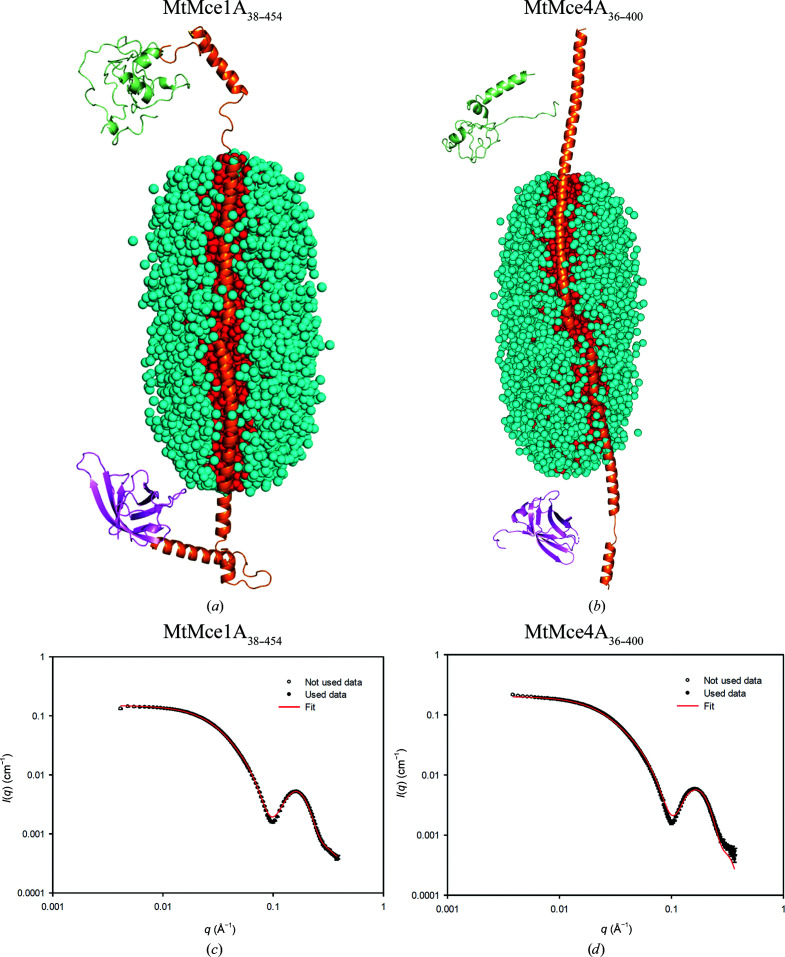
The extended helical models of (*a*) MtMce1A_38–454_ and (*b*) MtMce4A_36–400_ interacting with the core of the DDM micelle. The DDM molecules are represented as spheres and the protein as a cartoon. The model is represented by three domains: the MCE domain, helical domain and tail domain. The MCE domains of MtMce1A_38–454_ and MtMce4A_36–400_ are shown in magenta. The helical and tail domains are represented in orange and green, respectively, in both models. The DDM core and shell are shown in red and cyan, respectively. The MCE and the tail domains are not fully connected to the helical domain, as we used soft restraints between the three domains while fitting the model to the SAXS data to allow some flexibility. (*c*, *d*) The fits (red line) of the experimental SAXS data (black dots) to the proposed models of (*c*) MtMce1A_38–454_ and (*d*) MtMce4A_36–400_ are shown.

The crystallographic data for the *Mtb* MCE domain suggest that the *Mtb* SBPs cannot assemble as a homohexameric complex. In order to obtain greater insight into the possible structural properties, models of MtMce1A and MtMce4A were generated using *I-TASSER*. In both the MtMce1A and MtMce4A models, the extended helix of the helical domain turns back at residues Glu248 and Asp215, respectively, to form a coiled-coil structure, where the coiled-coil helices are held together by hydrophobic interactions. This brings the tail domain close to the MCE domain. This model is referred to as a coiled-coil model [Figs. 6 ▸(*a*) and 6 ▸(*b*)]. In addition, a second variation of this model was generated by opening the helical domain to form an extended helix, keeping the tail domain far away from the MCE domain. This model is referred to as an ‘extended helical model’ [Figs. 7 ▸(*a*) and 7 ▸(*b*)].

From our experimental data, it is clear that the MCE domain is soluble and the presence of the helical domain requires detergent for purification. Therefore, the detergent micelle has to interact with the helical domain. Although the helical domain has a high number of hydrophobic amino acids, it also has polar residues which preclude the possibility of the helix being completely inserted into the micelle, as for a typical transmembrane protein. Furthermore, calculations of the SAXS intensity with the helix inserted into the core show that the SAXS intensity for these models smears out the minimum, so that it is not as deep as observed in the data. Therefore, a core-shell model of the detergent micelle was used where the core represents the hydrophobic tail (dodecyl chains) of the detergent molecules and the shell represents the head group (polar) and the water molecules associated with it (Kaspersen *et al.*, 2014 ▸). The core-shell model of the detergent molecules is represented by Monte Carlo points acting as space holders for electron-density difference. On testing multiple micelle shapes using Monte Carlo points, the best fit was obtained when using a super-ellipsoid shape with the long axis along the helical domain, which maximizes the interaction of the protein helix with the core of the micelle. The micelle size (aggregation number) was initially estimated from SEC-MALS and SAXS scattering analysis (Kaspersen *et al.*, 2014 ▸) to be in the range 125–200. However, in cases where the fits were not satisfactory it was further varied in a reasonable range to obtain the best fit to the SAXS data.

With these assumptions, both coiled-coil as well as extended helical models for each of the MtMce1A and MtMce4A constructs were optimized (ten independent runs) together with the micelle with an appropriate aggregation number to fit the SAXS data. For MtMce1A_38–325_ as well as MtMce4A_39–320_ (MCE+Helical domains), both the coiled-coil and extended models showed convincing fits, with χ^2^ values ranging from 3 to 20 (Supplementary Figs. S15 and S16).

In the case of MtMce1A_38–454_ (MCE+Helical+Tail domains) the extended helical model [Fig. 7 ▸(*a*)] has a χ^2^ range of 15–24, compared with 22–50 for the coiled-coil model. The extended model fits the data well in the full *q* range, with a small deviation around the minimum, where the model curve is not quite low enough [Fig. 7 ▸(*c*)]. The coiled-coil model is too small, with some deviations at low *q*, and the optimization compensates partly by displacing the MCE domain away from the Helical+Tail domain, leading to some disconnectivity in the structure [Fig. 6 ▸(*a*)]. In the case of MtMce4A_36–400_ the data are not fitted well at high *q* values for both models, although the low-*q* data fit better to the extended model [Fig. 7 ▸(*d*)]. Similar to the MtMce1A_38–454_ coiled-coil model [Fig. 6 ▸(*a*)], the MCE domain and the Helical+Tail domain also become disconnected in the MtMce4A_36–400_ coiled-coil model [Fig. 6 ▸(*b*)].

The Helical+Tail domain fits for the MtMce4A_121–400_ extended and coiled-coil models have similar χ^2^ values in the range 4.6–8.0 (Supplementary Fig. S17). Both of these models have less deep minima with respect to the data. The MtMce1A_126–454_ extended model fitting has a χ^2^ range of 40–75, whereas the coiled-coil model fit shows a χ^2^ of between 114 and 182. Similar to the MtMce4A_121–400_ models, the minima are also less deep in the MtMce1A_126–454_ models (Supplementary Fig. S18). We have to accept that the tail domain is unstructured, with greater uncertainty in the structure prediction. This could also be a reason for the poorer SAXS fits for all of the constructs with the tail domain. The counting statistics of the data for the samples vary somewhat and therefore the χ^2^ values also vary, and it is observed that the χ^2^ values are often higher for data with good counting statistics. Therefore, we decided to also calculate *R* factors and weighted *R* factors, as used in crystallography. *R* factors are dominated by the high intensities at low *q*, whereas weighted *R* factors are a normalized measure of (χ^2^)^0.5^. The determined values are both in the range 1–5% (Supplementary Tables S5 and S6). They reveal that the deviation between data and fits is lower than the χ^2^ values suggest.

The above analysis confirms that the detergent micelle interacts with the helical domain irrespective of whether the helix turns back (as in a coiled-coil model) or is extended (as in an extended helical model) in MtMce1A and MtMce4A. High-resolution information is needed to unambiguously conclude which of these two models is relevant under physiological conditions. Considering the low-resolution information in the SAXS data, as well as the possible errors in the generated MtMce1A and MtMce4A models, which are partly based on structural predictions, our analysis gives the best possible explanation for the observed SAXS data in a qualitative and in a semi-quantitative manner. The methods applied here for the analysis of MtMce1A and MtMce4A can be generalized for use for other membrane proteins as well as for membrane-associated proteins purified in the presence of detergents.

## 4. Concluding remarks   

The challenges in purifying mycobacterial Mce proteins have hampered their study for many years. However, in this study recombinantly expressed and purified *Mtb* Mce1A–1F and Mce4A–4F SBPs have been characterized. Each of the SBPs was individually expressed and purified from *E. coli*. Further, we have classified the *Mtb* Mce1A–1F and Mce4A–4F SBPs into four different domains based on secondary-structure prediction. The domain characterization shows the presence of a unique tail domain in the SBPs from *Mtb* that is not present in the other characterized homologs [Fig. 1 ▸(*a*)]. The predicted length of the tail domain varies from 34 residues to 218 residues in the MtMce1A–1F and MtMce4A–4F SBPs. Further characterization shows that the full length as well as all of the domains of MtMce1A and MtMce4A remain as monomers in solution when purified individually. Only the MCE domain is soluble in the absence of detergents. The MCE domains of MtMce1A and MtMce4A occur as monomers in solution, as also shown by mass spectrometry. The crystal structure of the Mtb MCE domain reveals a β-barrel fold, as also found for its homologs, despite very low sequence identity (15% or less). The MCE+Helical and the Helical+Tail domain constructs require detergents for solubility. Further, SAXS analysis of MtMce1A, MtMce4A and their domains suggests that the helical domain may adopt the ‘coiled-coil’ or ‘extended helical’ conformation. In the coiled-coil model the MCE and tail domains are near each other, whereas the MCE and tail domains are far away from each other in the extended helical model. Irrespective of the conformation of the helical domain, it is very clear that the helical domain requires detergent for its stability and is either involved in interaction with the lipid substrates or embedded in the membrane. Structural analysis of MtMce4A_39–140_ suggests that the homohexamer cannot be formed, at least in Mce4A, due to multiple steric clashes. The fact that there are six Mce SBPs in *Mtb* suggests that the six MceA–F SBPs may interact with each other to form heterohexamers, where the helical domains of the six MceA–F molecules may form a channel as observed in EcPqiB (Ekiert *et al.*, 2017 ▸), but in *Mtb* this channel will be more extended. The resulting heterohexameric arrangement would therefore favor a tunnel-based mechanism for lipid transport. The presence of a single MCE domain, a longer helical domain and an additional tail domain will make the overall architecture of the mycobacterial Mce complexes different from other recently characterized Mce homologs. The studies reported here provide a good base for future high-resolution studies of the MtMceA–F SBPs and the entire Mce complexes to further understand the detailed structural arrangement as well as the lipid-transport mechanism of the mycobacterial Mce complexes.

## 5. Abbreviations   

The abbreviations used are as follows. Ab, *Acetinobacter baumannii*; CD, circular dichroism; C_12_E_9_, dodecyl nona­ethylene glycol ether; DDM, *n*-dodecyl β-d-maltoside; Ec, *Escherichia coli*; FC-12, Fos-choline-12; HEPES, 4-(2-hydroxyethyl)piperazine-1-ethanesulfonic acid; IM, inner membrane; IPTG, isopropyl β-d-1-thiogalactopyranoside; MCE, mammalian cell entry; MES, 2-(*N*-morpholino)ethanesulfonic acid; MlaD, membrane lipid asymmetry; PDC, protein–detergent complex; PqiB, paraquat-inducible protein B; LetB, lipophilic envelope-spanning tunnel B; MS, mass spectrometry; *Mtb*, *Mycobacterium tuberculosis*; OM, outer membrane; SAD, single-wavelength anomalous dispersion; SBP, substrate-binding protein; SAXS, small-angle X-ray scattering; SEC, size-exclusion chromatography; SeMet, selenomethionine; Tb, tuberculosis; Tris, tris(hydroxymethyl)aminomethane.

## Supplementary Material

PDB reference: MtMce4A_39–140,_ native, 7ai3

PDB reference: SeMet-labelled, 7ai2

SASBDB reference: MtMce1A_36–148_, SASDJU9

SASBDB reference: MtMce1A_38–454_, SASDK32

SASBDB reference: MtMce1A_126–454_, SASDK22

SASBDB reference: MtMce1A_38–325_, SASDJZ9

SASBDB reference: MtMce4A_39–140_, SASDJV9

SASBDB reference: MtMce4A_36–400_, SASDJW9

SASBDB reference: MtMce4A_121–400_, SASDJX9

SASBDB reference: MtMce4A_39–320_, SASDJY9

Supplementary Results, Figures and Tables. DOI: 10.1107/S2052252521006199/mf5054sup1.pdf

SASBDB data for the relevant Mce domains.: https://www.sasbdb.org/project/1178/q9vh6j46wj/

## Figures and Tables

**Table 1:** Crystallization, data-collection and refinement statistics for the MtMce4A_39–140_ structures Values in parentheses are for the highest resolution shell.

Data set	SeMet-labeled	Native
Crystallization		
Protein storage buffer	50 m*M* MOPS, 350 m*M* NaCl, 10% glycerol pH 7.0	50 m*M* MOPS, 350 m*M* NaCl, 10% glycerol, 5 m*M* DTT pH 7.0
Protein concentration (mg ml^−1^)	7.5	7.5
Well solution buffer	100 m*M* MES, 700 m*M* ammonium sulfate pH 6.0	100 m*M* sodium HEPES, 100 m*M* LiCl_2_, 20% PEG 400 pH 7.5
Cryoprotectant buffer	25% glycerol, 100 m*M* MES, 700 m*M* ammonium sulfate pH 6.0	20% ethylene glycol, 100 m*M* sodium HEPES, 100 m*M* LiCl_2_, 20% PEG 400 pH 7.5
Temperature (°C)	22	22
Data collection		
Beamline	BioMax, MAX IV	BioMax, MAX IV
Wavelength (Å)	0.968	0.953
Detector	EIGER 16M hybrid pixel	EIGER 16M hybrid pixel
Detector distance (mm)	357.46	276.71
Oscillation range (°)	0.1	0.1
Data processing		
Space group	*P*6_5_	*P*6_5_
*a*, *b*, *c* (Å)	134.0, 134.0, 105.5	131.2, 131.2, 105.5
α, β, γ (°)	90, 90, 120	90, 90, 120
Resolution range (Å)	48–3.6 (3.9–3.6)	47.8–2.9 (3.0–2.9)
*R*_p.i.m._	0.11 (1.45)	0.06 (0.79)
Multiplicity	12.4 (12.5)	15.4 (15.6)
Wilson *B* factor (Å^2^)	108.4	66.3
Solvent content (%)	72.9	71.7
Total No. of reflections	155214 (36878)	355222 (57858)
No. of unique reflections	12505 (2961)	23016 (3714)
CC_1/2_ (%)	99.6 (23.0)	99 (43.6)
〈*I*/σ(*I*)〉	7.1 (1.2)	11 (1.4)
Completeness (%)	99.8 (99.5)	100 (99.9)
Refinement statistics		
*R*_work_	0.2165	0.1947
*R*_free_	0.2466	0.2348
No. of atoms		
Protein	3271	3276
Water	—	14
Average *B* factor (Å^2^)		
Protein	158.9	92.2
Water	—	79.1
Ramachandran statistics		
Favored (%)	93.8	97.0
Allowed (%)	6.0	3.0
Outliers (%)	0.2	0.0
R.m.s. deviations		
Bond lengths (Å)	0.002	0.002
Bond angles (°)	0.560	0.422
PDB code	7ai2	7ai3

**Table 2:** Molecular masses of MtMce1A and MtMce4A domains as calculated from SEC-MALS

Protein name	Theoretical monomeric molecular mass (kDa)	Protein–DDM conjugate (SEC-MALS) (kDa)	Protein (SEC-MALS) (kDa)	Empty DDM micelle (SEC-MALS) (kDa)
MtMce1A_36–148_	16.9	—	16.4	—
MtMce1A_38–325_	36.0	103.0	39.0	66.0
MtMce1A_126–454_	38.7	144.0	40.0	58.0
MtMce1A_38–454_	48.3	159.0	58.0	68.0
MtMce4A_39–140_	15.0	—	15.0	—
MtMce4A_39–320_	34.4	102.5	34.9	67.5
MtMce4A_121–400_	34.6	128.9	44.7	65.1
MtMce4A_36–400_	43.5	142.0	55.4	70.1
