# Supplementary material for: Structural insights into the substrate-binding proteins Mce1A and Mce4A from Mycobacterium tuberculosis
Source: IUCrJ. 2021 Jul 28;8(Pt 5):757–74. doi: 10.1107/S2052252521006199 (PMC8420772; doi:10.1107/S2052252521006199)
Supplement: Supplementary file 1 [file m-08-00757-sup1.pdf]

IUCrJ

Volume 8 (2021)

Supporting information for article:

Structural insights into the substrate-binding proteins Mce1A and Mce4A from *Mycobacterium tuberculosis*

Pooja Asthana, Dhirendra Singh, Jan Skov Pedersen, Mikko J. Hynönen, Ramita Sulu, Abhinandan V. Murthy, Mikko Laitaoja, Janne Jänis, Lee W. Riley and Rajaram Venkatesan

S1. Supplementary Results

S1.1. Recombinant expression and purification of MtMce1A-1F proteins

Initially, the full length MtMce1A-1F were cloned and their expression in *E. coli* tested individually which showed that all the MtMce1A-1F individual proteins were successfully expressed. From these, MtMce1A, MtMce1B, MtMce1C and MtMce1F were selected for further purification trials as the expression for MtMce1D and MtMce1E was very low. Given that, all the Mce SBPs have N-terminal transmembrane domain, their solubility was assayed in buffers containing detergents such as DDM, Fos-choline-12 (FC-12) and Dodecyl nonaethylene glycol ether ($C_{12}E_9$) as mentioned in the methods section. Among these MtMce1A, MtMce1C, and MtMce1F were purified in the presence of DDM, whereas MtMce1B was purified only in the presence of FC-12. The major peak for the eluted proteins were between 10 and 12 mL in a 24 mL Superdex 200 10/300 column. An additional 8 ml peak was observed, suggesting the presence of soluble aggregates even in the presence of detergents.

Subsequently, the transmembrane domain deleted constructs were generated for MtMce1A and, MtMce1B (MtMce1A_{38-454} and MtMce1B_{29-346}) to test if they could be purified without detergents. Surprisingly, even after deleting the transmembrane region, the proteins still required detergents for their purification (Fig. S3A). Also, as the expression of MtMce1E was very less, TM domain deleted construct was also made for MtMce1E (MtMce1E_{37-390}). Interestingly, the deletion resulted in enhanced expression of MtMce1E_{37-390} when compared to the full length MtMce1E. However, for this construct a combination of DDM and $C_{12}E_9$ detergent was needed in different steps to purify it further (Supplementary Table 3). The TM domain deleted MceA-F SBPs almost eluted in the same volume as the full length MtMce1A-1F proteins. As the deletion of TM domain did not preclude the use of detergent, it was not generated for MtMce1C. However, in case of MtMce1F recombinant protein expression, heavy degradation was observed. We predicted that the degradation could mainly be at the extended and unstructured tail domain. The tail domain of Mce1D is also long and has similarity with the tail domain of Mce1F. Therefore, transmembrane domain and tail domain deleted shorter constructs for MtMce1D (MtMce1D_{44-314}) and MtMce1F (MtMce1F_{30-314}) were generated and these constructs showed significantly less degradation (Fig. S3A). Intriguingly, even these constructs could be purified only in the presence of detergents.

S1.2. Recombinant expression and purification of MtMce4A-4F proteins

In parallel, full-length MtMce4A-4F individual constructs were successfully generated in *E. coli.* Expression tests showed that the MtMce4F had the least expression. After initial detergent screening, MtMce4A, MtMce4C, MtMce4D and MtMce4F were purified in buffers containing the detergent DDM, whereas MtMce4B and MtMce4E required FC-12 for their purification. Gel filtration profile of MtMce4A, MtMce4C and MtMce4F showed that the aggregated protein peak (8 mL) was well separated from protein-detergent peak (~12 mL). In case of MtMce4B, MtMce4D and MtMce4E the aggregated protein and protein-detergent complex (PDC) eluted together in one broad peak. MtMce4B, MtMce4D and MtMce4F showed heavy degradation on SDS PAGE (Fig. S3B). As similar degradation was also observed for MtMce1F, it is possible that also in MtMce4B, MtMce4D and MtMce4F the degradation could be in the unstructured tail domain.

S1.3. Conformational changes in MtMce1A$_{36\text{-}148}$ and MtMce4A$_{39\text{-}140}$

Comparison of the secondary structure content of MtMce4A$_{39\text{-}140}$ calculated from the CD spectrum with the crystal structure showed higher β sheet content (39%) in crystal than from the experimental CD spectra (28%) data (Table S10). The result indicate that the protein is more structured in the crystallization condition and attain the MCE β-barrel fold. Interestingly, during thermal melting analysis when the temperature was gradually increased from 22 °C to 92 °C, a broad shift in the peak was observed between 220-240 nm corresponding to increase in secondary structures (Fig. S9A). This also aligned with the BeStSel analysis, which showed an increase in the β-sheet content with increasing temperatures. The β-sheet content at 72 °C was slightly higher (33%) indicating the unusual property of heat-induced conformational change of MtMce4A$_{39\text{-}140}$. However, the data after 72 °C was not reliable due to poor fitting of the data at 190-200 nm. Besides, the peak shift disappeared upon cooling of MtMce4A$_{39\text{-}140}$ indicating the temperature dependent reversible nature of this conformational change (Fig. S9B).

Similar thermal melting analysis of MtMce1A$_{36\text{-}148}$ showed conformational change upon heating. The initial peak at 198 nm, shifted to a broader range of 205-230 nm when the protein was heated from 22 °C to 92 °C (Fig. S8A). Moreover, the peaks at 210-230 nm were stable (not reversible) when the sample was recooled, whereas the peak between 205-210 nm disappeared during recooling (Fig. S8B). By visually looking at the spectra at 22 °C and 92 °C, one can interpret that MtMce1A also attains more secondary structure upon heating as observed in MtMce4A$_{39\text{-}140}$. Intriguingly, the deconvolution analysis (200-250 nm) of these peaks

for MtMce1A$_{36-148}$ in both CDNN and BeStSel software suggested that only the spectra at 22 °C have higher β-sheet content and the β-sheet content reduced gradually upon heating. This overall indicates the challenges in the interpretation of CD spectra of β-sheet rich proteins even with the best available programs. In any case, we can clearly see that both MtMce1A$_{36-148}$ and MtMce4A$_{39-140}$ undergoes conformational change upon heating. It is possible that in the purified conditions, both MtMce1A$_{36-148}$ and MtMce4A$_{39-140}$ are in non-native conformations and MtMce4A$_{39-140}$ attains native conformation in the crystallization buffer.

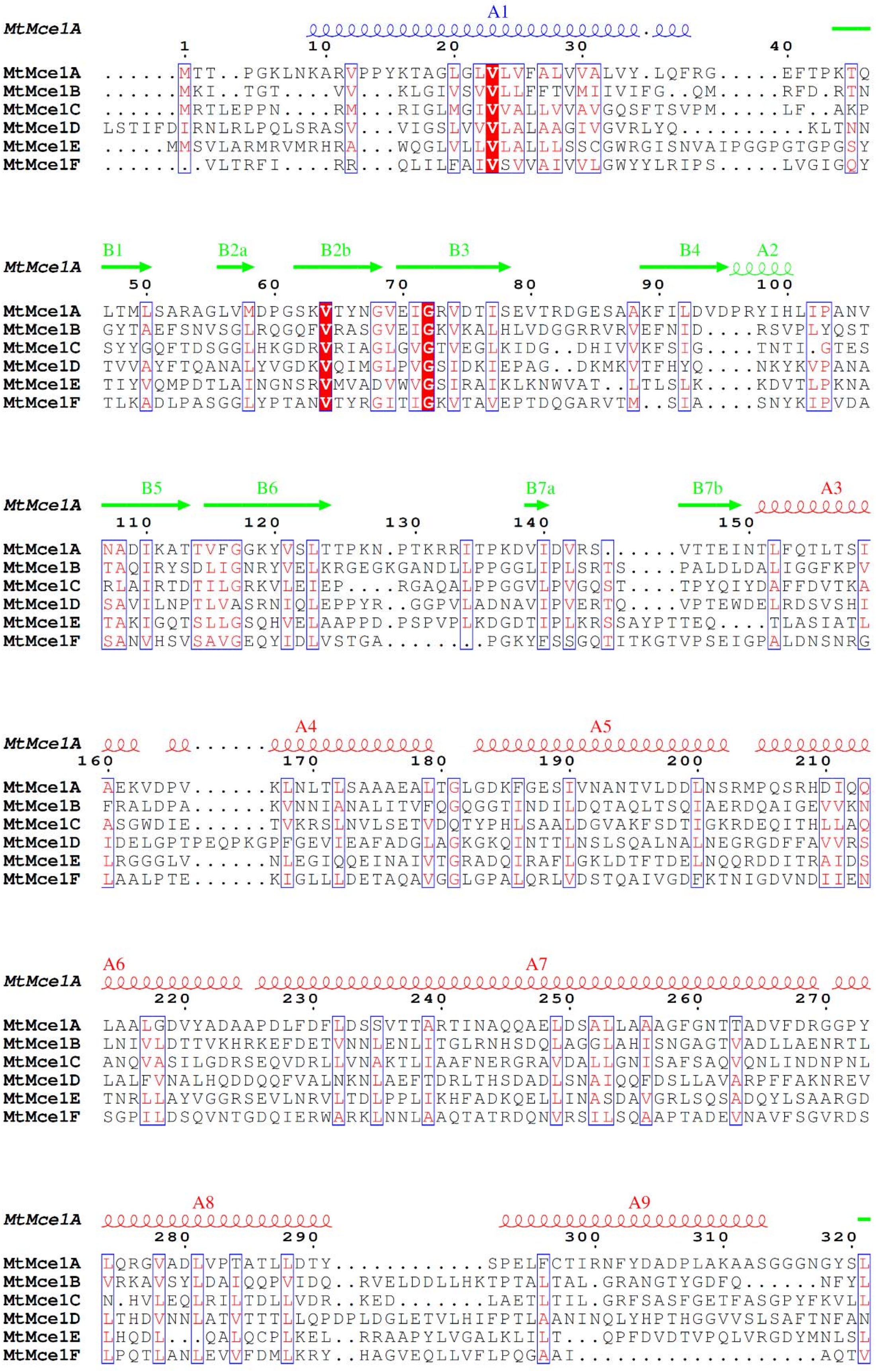
A1
B1 B2a B2b B3 B4 A2
MtMce1A LTMLSARAGLVMDPGSKVTYNGVEIGRVDTISEVTRDGESAAKFILDVDPRYIHLIPANV
MtMce1B GYTAEFSNVSGLRQGQFVRASGVEIGKVKALHLVDGGRRVRVEFNID....RSVPLYQST
MtMce1C SYYGQFTDSGGLHKGDRVRIAGLGVGTVEGLKIDG..DHIVVKFSIG....TNTI.GTES
MtMce1D TVVAYFTQANALYVGDKVQIMGLPVGSIDKIEPAG..DKMKVTFHYQ....NKYKVPANA
MtMce1E TIYVQMPDTLAINGNSRVMVADVWVGSIRAIKLKNWVAT..LTLSLK....KDVTLPKNA
MtMce1F TLKADLPASGGLYPTANVTYRGITIGKVTAVEPTDQGARVTM..SIA....SNYKIPVDA
MtMce1A ......MTT..PGKLNKARVPPYKTAGLGLVLVFALVVALVY.LQFRG.....EFTPKTQ
MtMce1B ......MKI..TGT....VV...KLGIVSVVLLFFTVMIIVIFG..QM.....RFD.RTN
MtMce1C ......MRTLEPPN....RM...RIGLMGIVVALLVVAVGQSFTSVPM.....LF..AKP
MtMce1D LSTIFDIRNLRLPQLSRASV...VIGSLVVVLALAAGIVGVRLYQ.........KLTNN
MtMce1E .....MMSVLARMRVMRHRA...WQGLVLLVLALLLSSCGWRGISNVAIPGGPGTGPGSY
MtMce1F .......VLTRFI....RR...QLILFAIVSVVAIVVLGWYYLRIPS.....LVGIGQY
B5 B6 B7a B7b A3
MtMce1A NADIKATTVFGGKYVSLTTPKN.PTKRRITPKDVIDVRS......VTTEINTLFQTLTSI
MtMce1B TAQIRYSDLIGNRYVELKRGEGKGANDLLPPGGLIPLSRTS....PALDLDALIGGFKPV
MtMce1C RLAIRTDTILGRKVLEIEP....RGAQALPPGGVLPVGQST....TPYQIYDAFFDVTKA
MtMce1D SAVILNPTLVASRNIQLEPPYR..GGPVLADNAVIPVERTQ....VPTEWDELRDSVSHI
MtMce1E TAKIGQTSLLGSQHVELAAPPD.PSPVPLKDGDTIPLKRSSAYPTTEQ....TLASIATL
MtMce1F SANVHSVSAVGEQYIDLVSTGA.......PGKYFSSGQTITKGTVPSEIGPALDNSNRG
A4 A5
MtMce1A AEKVDPV......KLNLTLSAAAEALTGLGDKFGESIVNANTVLDDLNSRMPQSRHDIQQ
MtMce1B FRALDPA......KVNNIANALITVFQGQGGTINDILDQTAQLTSQIAERDQAIGEVVKN
MtMce1C ASGWDIE......TVKRSLNVLSETVDQTYPHLSAALDGVAKFSDTIGKRDEQITHLLAQ
MtMce1D IDELGPTPEQPKGPFGEVIEAFADGLAGKGKQINTTLNSLSQALNALNEGRGDFFAVVRS
MtMce1E LRGGGLV......NLEGIQQEINAIVTGRADQIRAFLGKLDTFTDELNQQRDDITRAIDS
MtMce1F LAALPTE......KIGLLLDETAQAVGGLGPALQRLVDSTQAIVGDFKTNIGDVNDIIEN
A6 A7
MtMce1A LAALGDVYADAAPDLFDFLDSSVTTARTINAQQAELDSALLAAAGFGNTTADVFDRGGPY
MtMce1B LNIVLDTTVKHRKEFDETVNNLENLITGLRNHSDQLAGGLAHISNGAGTVADLLAENRTL
MtMce1C ANQVASILGDRSEQVDRLLVNAKTLIAAFNERGRAVDALLGNISAFSAQVQNLINDNPNL
MtMce1D LALFVNALHQDDQQFVALNKNLAEFTDRLTHSDADLSNAIQQFDSLLAVARPFFAKNREV
MtMce1E TNRLLAYVGGRSEVLNRVLTDLPPLIKHFADKQELLINASDAVGRLSQSADQYLSAARGD
MtMce1F SGPILDSQVNTGDQIERWARKLNNLAAQTATRDQNVRSILSQAAPTADEVNAVFSGVRDS
A8 A9
MtMce1A LQRGVADLVPTATLLDTY...........SPELFCTIRNFYDADPLAKAASGGGNGYSL
MtMce1B VRKAVSYLDAIQQPVIDQ..RVELDDLLHKTPTALTAL.GRANGTYGDFQ......NFYL
MtMce1C N.HVLEQLRILTDLLVDR..KED.......LAETLTIL.GRFSASFGETFASGPYFKVLL
MtMce1D LTHDVNNLATVTTTLLQPDPLDGLETVLHIFPTLAANINQLYHPTHGGVVSLSAFTNFAN
MtMce1E LHQDL...QALQCPLKEL..RRAAPYLVGALKLILT...QPFDVDTVPQLVRGDYMNLSL
MtMce1F LPQTLANLEVVFDMLKRY..HAGVEQLLVFLPQGAAI..................AQTV

```
          B8    B9        A10      A11                                   A12
MtMce1A
                   330              340                 350                 360
MtMce1A   RTNSEILSGIGISLLSPLALATN....GAAIGIGLVAG...LIAPPLAVAAN.......L
MtMce1B   .................CDL..................................QIK..W
MtMce1C   .................ANLVPG..................QILQPFVD....AAFK..K
MtMce1D   ................PMEFICSSIQAGSRLGYQESAELCAQYLAPVLDAIKFNYFPFGL
MtMce1E   ................TLDLTYS..................AIDNA........FLT..G
MtMce1F   .................LTPTPG..................AAQLPLAPAINYPPPC..L

                                                                   B10
MtMce1A
          370          380          390          400          410          420
MtMce1A   AGALPG...IVGGAPNPYTYPEN.LPRVNARGGPGGAPGCWQPITRDLWPAPYLVMDTGA
MtMce1B   NGFQAG......GPVRT....VKLFSQPTGRCTP...Q......................
MtMce1C   RGISPE......DFWRSAGLPAYRWPDPNGTRFP...NGAPPP........PPPVLEGTP
MtMce1D   NVASTA.....STLPKEIAYSEPRLQPPNGYKDT.TVPGIWVP........DTPLSHR..
MtMce1E   TGFSGALRALEQSFGRDP...ETMIPD..IRYTP...NPNDAP........GGPLVERG.
MtMce1F   TGFLPA......SEWRSPADTSP.....................................

                                         A13
MtMce1A
                430                            440          450
MtMce1A   SLAPYNHMEVGSPY.................AVEYVWGRQVGDNTIN.............
MtMce1B   ............................................................
MtMce1C   E.HPGPAVPPGSPCSYTPPADGLPRPWDPLPCANLTQGPFGG..................
MtMce1D   .....................NTQPGWVVAPGMQGV...QVGPITQGLLTPESLAELM..
MtMce1E   ..........NRQC..............................................
MtMce1F   .....RPLPSGTYCKIPQDAQLQVRGARNIPCVDVLGKRAATPKEC..RSKDPYVPLGTN

MtMce1A

MtMce1A   ............................................................
MtMce1B   ............................................................
MtMce1C   PDFPAPLDVATSP..PNPDGPPPAPGLPIAGRPG.....EVPPNVPGTPVPIPQE.APP
MtMce1D   ...............GGPDIAPPSSGLQTPPGPPN.........AYDEYPV.........
MtMce1E   ............................................................
MtMce1F   PWFGDPNQILTCPAPGARCDQPVKPGLVIPAPSINTGLNPAPADQVQGTPPPVSDPLQRP

MtMce1A

MtMce1A   ............................................................
MtMce1B   ............................................................
MtMce1C   GARTL.PLG.PAPGPAPPPAAPGPPAPPGPGPQLPAPFINPGGTGGS...GVTGGSEN..
MtMce1D   ....LPPIGLQAPQVPIPPPPPGPDVIPGPVPPTPAPVGAPLPA......EAGGGQ....
MtMce1E   ............................................................
MtMce1F   GSGTVQCNG.QQPNPCVYTPTSGPSAVYSPA...SGELVGPDGVKYAVANSSTTGDDGWK

MtMce1A

MtMce1A   .......
MtMce1B   .......
MtMce1C   .......
MtMce1D   .......
MtMce1E   .......
MtMce1F   EMLAPAS
```

Figure S1 Multiple sequence alignment of MtMce1A-1F. The secondary structure elements of the MCE domain are based on the crystal structure of MtMce4A$_{39\text{-}140}$ and the remaining domains are from secondary structure prediction.

```
                                         A1                          B1
MtMce4A
                      1       10        20        30        40
MtMce4A  ............MS.GGGSRRTSVRVAAALLAGLMVGSAVLTYLSYTAAFTSTDTVTVSS
MtMce4B  MAGSG.VPSHRSMVIKVS....VFAVVMLLVA.AGLVVVFGD......FRFGPTTVYHAT
MtMce4C  MLNRKPSSKHERDPLRTGIFGLVLVICVVLIA.F...GYSGL......PFWPQGKTYDAY
MtMce4D  MMG........RVAMLTGSRGLRYATVIALVA.ALVGGVYVL......SSTGNKRTIVGY
MtMce4E  ............MN.RIWLRAIILTASSALLAGCQFGGLNSLPLPGTAGHGEGAYSVTVE
MtMce4F  MIDR........LA.KIQLSIFAVITV....ITLSVMAIFYLRLPA..TFGIGTYGVSAD

            B2a      B2b      B3           B4      A2        B5
MtMce4A
          50        60        70        80        90        100
MtMce4A  PRAGLVMEKGAKVKYRGIQVGKVTDISYSGNQAR...LKLAIDSGEMGFIPSNATVRIAG
MtMce4B  FTDASRLKAGQKVRIAGVPVGSVKAVKLNP..D..HSIDVAFAIDRSYTLYSSTRAVIRY
MtMce4C  FTDAGGITPGNSVYVSGLKVGAVSAVSLAG.....NSAKVTFSVDRSIVVGDQSLAAIRT
MtMce4D  FTSAVGLYPGDQVRVLGVPVGEIDMIEPRS.....SDVKITMSVSKDVKVPVDVQAVIMS
MtMce4E  MADVATLPQNSPVMVDDVTVGSVAGIVAVQRPDGSFYAAVKLDLDKNVLLPANAVAKVSQ
MtMce4F  FVAGGGLYKNANVTYRGVAVGRVESVGL..NPNG...VTAHMRLNSGTAIPSNVTATVRS

              B6                    B7a   B7b       A3
MtMce4A
           110       120       130       140       150       160
MtMce4A  NTIFGAKSVEFIPPKTPSP.KPLSPNAHVAASQVQLEVNTLFQSLIDLLHKIDPLETNA.
MtMce4B  ENLVGDRFLEITSGPGE..LRKLPPG.....GTINVAHTQPALDLDALLGGLRPVLKGFD
MtMce4C  DTILGERSIAVSPAGSG..KS..........TTIPLSRTTTPYTLNGVLQDLGRNANDLN
MtMce4D  PNLVAARFIQLTPVYTG..GAVLPDN.....GRIDLDRTAVPVEWDEVKEGLTRLAADLS
MtMce4E  TSLLGSLHVELAPPTDRPPTGRLVDG.....SRITEANTDRFPTTEEVFSALGVVVNKGN
MtMce4F  VSAIGEQYIDLVPPEN.PSSTKLRNG.....FRIQRQNTRIGQDVADLLRQAETLLGSLG

             A4                      A5
MtMce4A  ............
                        170       180       190       200       210
MtMce4A  ............TLSALSEGLRGHGDDLGALLSGLNTLTRQANPKLPALQEDFRKAAVVA
MtMce4B  ADKIN......TITSAVIELLQGQGGPLANVLADTG.....................AFS
MtMce4C  RPQFE......QALNVF..........................................T
MtMce4D  PAAGELQGPLGAAINQAADTLDGNGDSLHNALRELA.....................QVA
MtMce4E  VGALE......EIIDETHQAVAGRQAQFVNLVPRLA.....................ELT
MtMce4F  DTRLR......ELLHEAFIATNGAGPELARLIESARLLVDEANANYPQVSQLIDQAGPFL

            A6                                                A7
MtMce4A
                220       230       240                 250
MtMce4A  NVYADAAGDLNTVFDNLPTINKTIVDQKDNLNDTLLA..............T...IGLSN
MtMce4B  AALGARDQLIGEVITNLNAV......................LATVDAKSAQFSASVDQLQ
MtMce4C  QALHDATPQVRGAVDGLTSLSRALNRRDEALQGLLAHAKSVTSVLSERAEQVNKLVEDGN
MtMce4D  GRLGDSRGDIFGTVKNLQVLVDALSESDEQIVQFAGHVASVSQVLADSSA..........
MtMce4E  AGLNRQVHDIIDALDGLNRVSAILARDKDNLGRALDT..............LPDAVRVLN
MtMce4F  QAQIRAGGDIKSLADGLARFTWQLRAADPRLRDTLAD..............APDAIDEAN

                                                A8
MtMce4A  ..............................
               260       270       280
MtMce4A  NAYETLAPAEQNFIDAINRLRAPLKVTSD...............................
MtMce4B  QLVSGLAKNRDPIAGAISPLASTTTDLTELLRNSRRPLQGILENARPLATELDNRKAEVN
MtMce4C  QLFAALDARRAALSALISGIDDVAAQISGFVADNRKEFGPALSKLNLVLANLNERRDYIT
MtMce4D  ..........NLDQTLGTLNQALSDIRGFLRENNSTLIETVNQLNDFAQTLSDQSENIE
MtMce4E  QNRDHI.......VDAFAALKRLTMVTSHVLAETKVDFGEDLKDLYSIVKALNDDRKDFV
MtMce4F  TAFSGI.......RPSFPALAASLA....NLGRVGVIYHKSIE.................
```

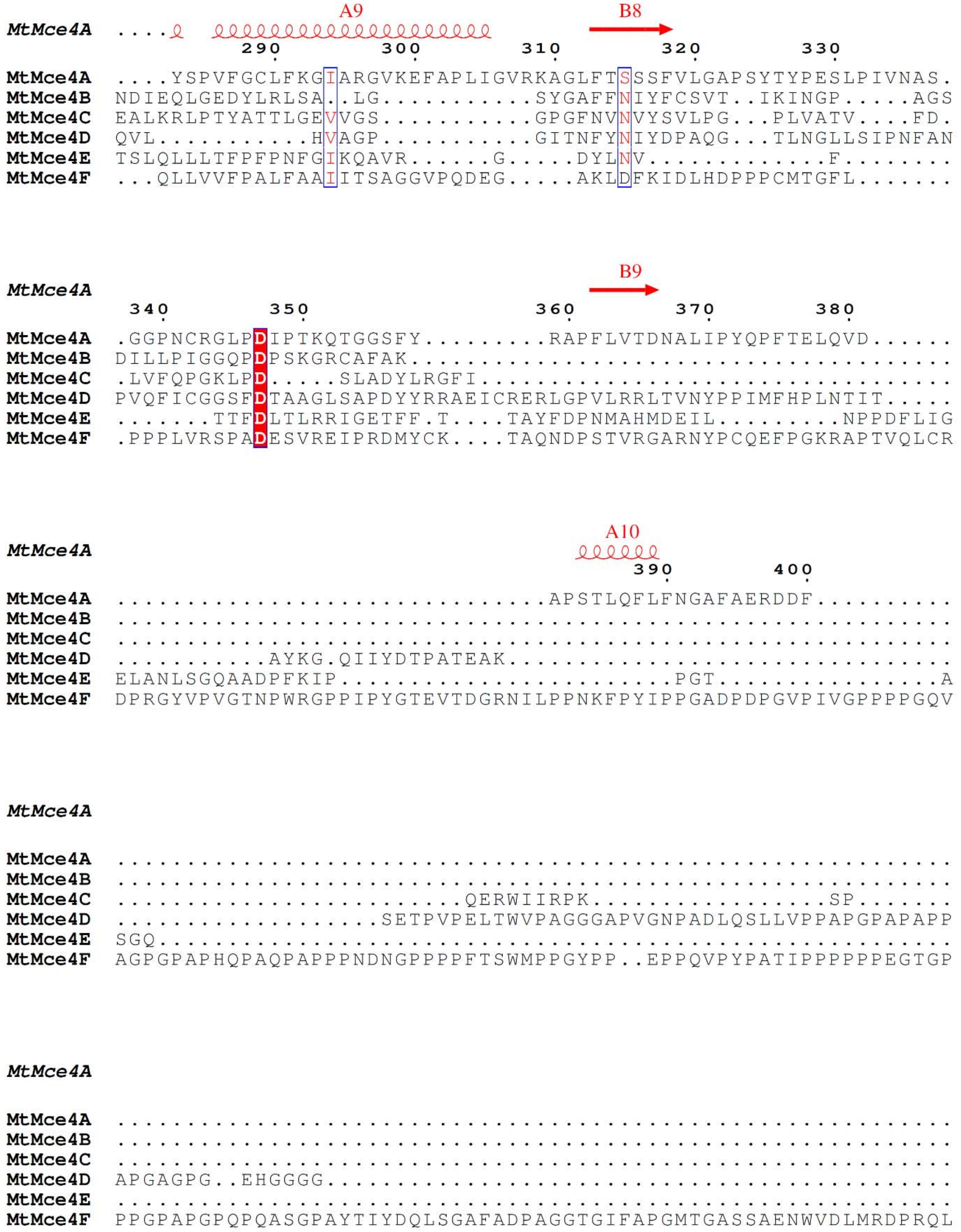

Figure S2 Multiple sequence alignment of MtMce4A-4F. The secondary structure elements of the MCE domain are from the crystal structure of MtMce4A$_{39\text{-}140}$ and the remaining domains are from secondary structure prediction.

(A)

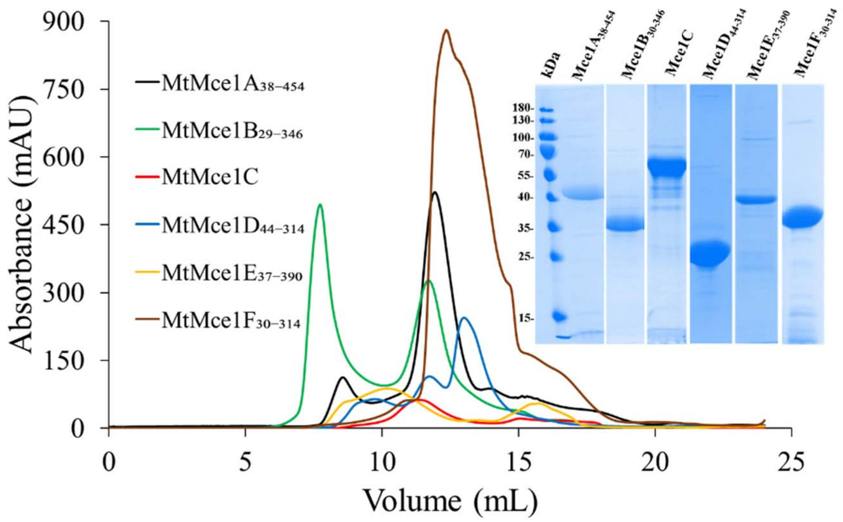

(B)

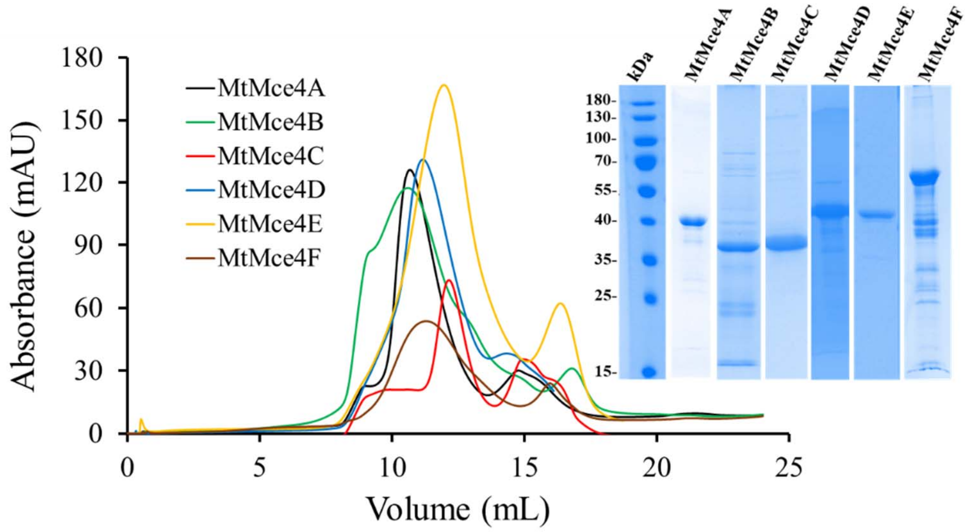

Figure S3 SEC elution profiles of (A) selected individual MtMce1A-1F SBPs and (B) individual MtMce4A-4F SBPs on a 24 mL Superdex 200 10/300 column. The protein samples were analyzed on a 12 % SDS-PAGE (inset).

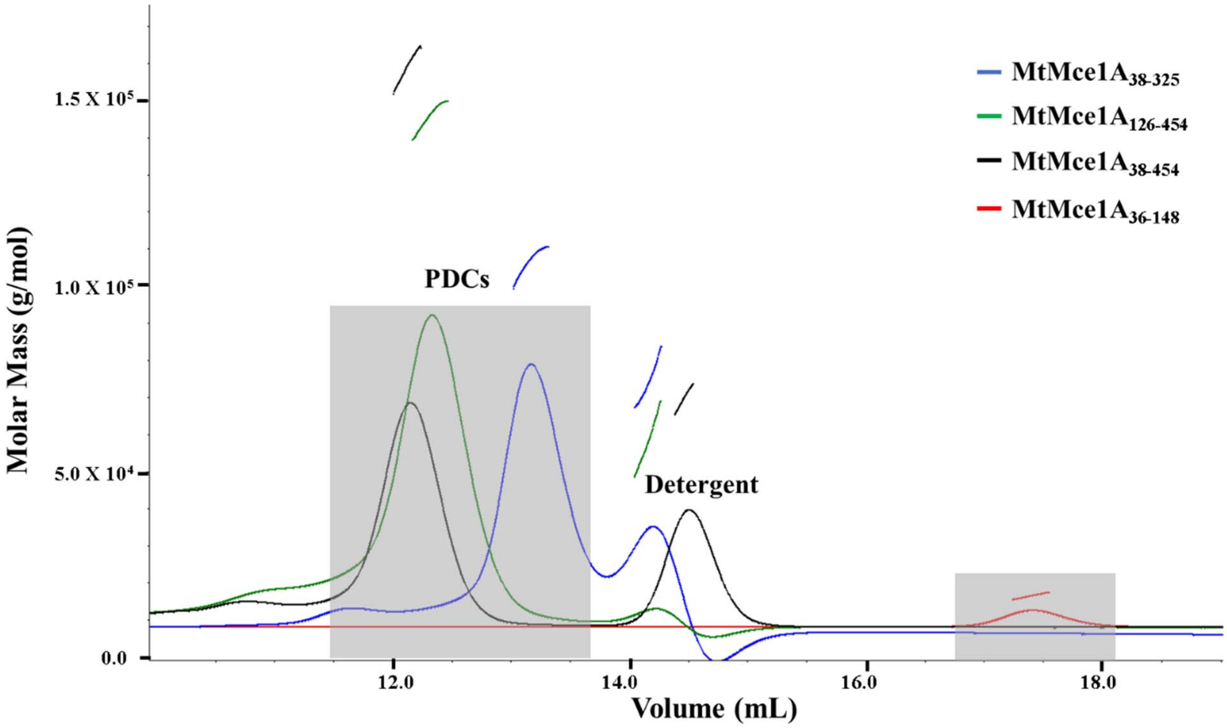

Figure S4 SEC-MALS profile of Mce1A$_{36\text{-}148}$ (red), Mce1A$_{38\text{-}325}$ (blue), Mce1A$_{126\text{-}454}$ (green), and Mce1A$_{38\text{-}454}$ (black). Mce1A$_{36\text{-}148}$ has a single scattering peak at ~17.5 mL. Whereas other Mce1A domains are purified in DDM showed two scattering peaks corresponds to protein-detergent complex (12-14 mL) and empty detergent micelle. All the samples were injected on a 24 mL Superdex 200 10/300 column.

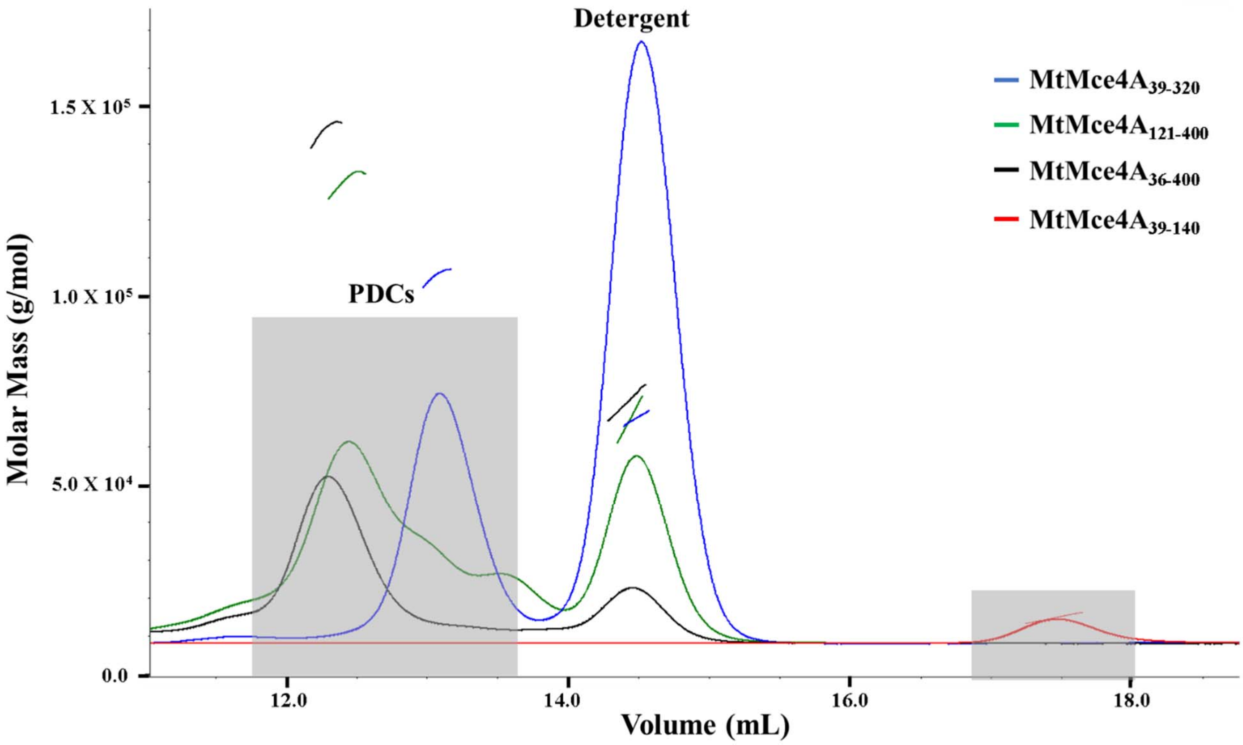

Figure S5 SEC-MALS profile of Mce4A$_{39-140}$ (red), Mce4A$_{39-320}$ (blue), Mce4A$_{121-400}$ (green), and Mce4A$_{36-400}$ (black). Mce4A$_{39-140}$ has a single scattering peak at ~17.5 mL. Whereas other Mce4A domains are purified in DDM showed two scattering peaks corresponds to protein-detergent complex (12-14 mL) and empty detergent micelle. All the samples were injected on a 24 mL Superdex 200 10/300 column.

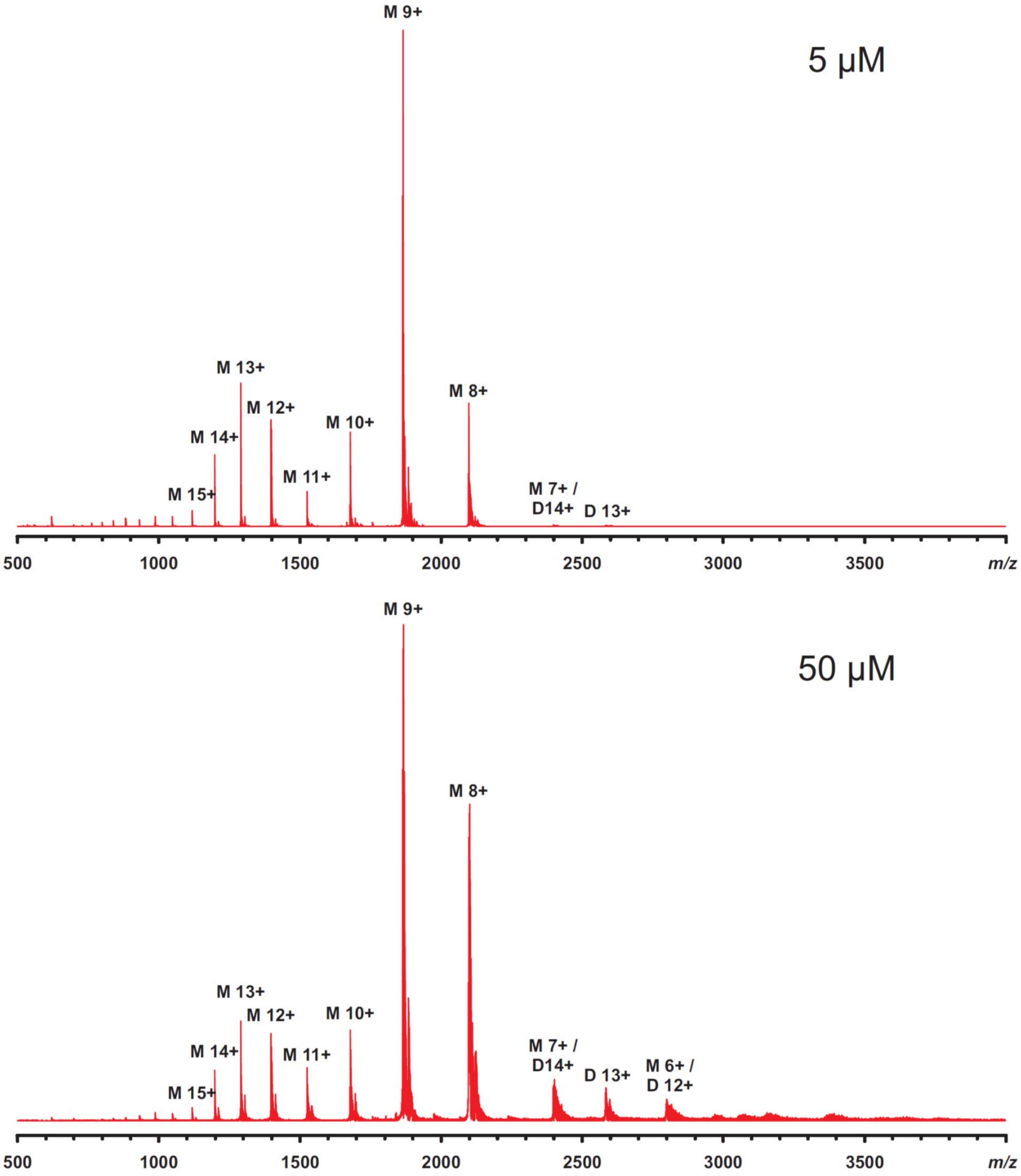

Figure S6 Native mass spectra of MtMce1A$_{36\text{-}148}$ at 5 µM and 50 µM concentration in 20 mM ammonium acetate buffer, pH 6.8.

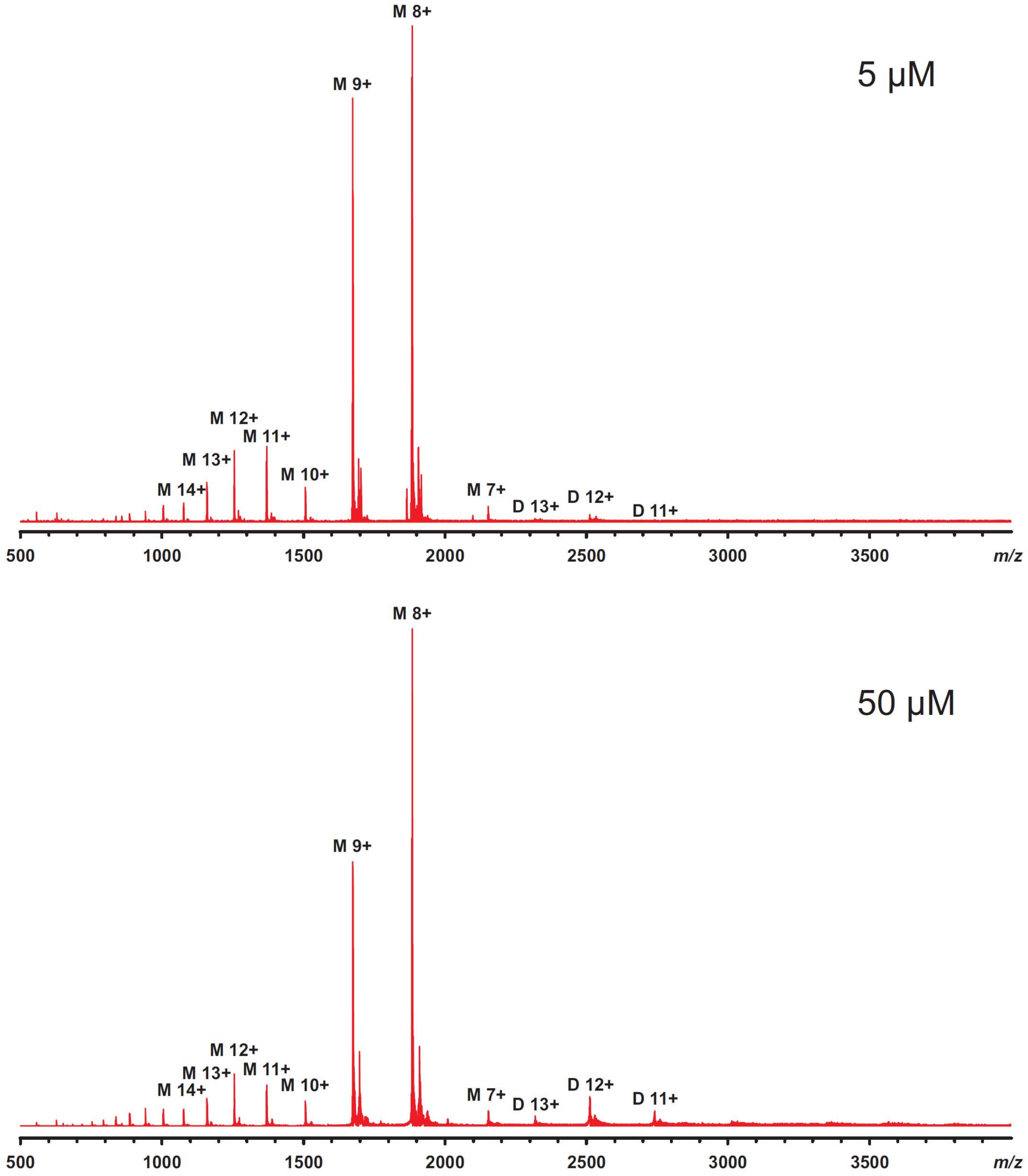

Figure S7 Native mass spectra of MtMce4A$_{39\text{-}140}$ at 5 µM and 50 µM concentration in 20 mM ammonium acetate buffer, pH 6.8.

(A)

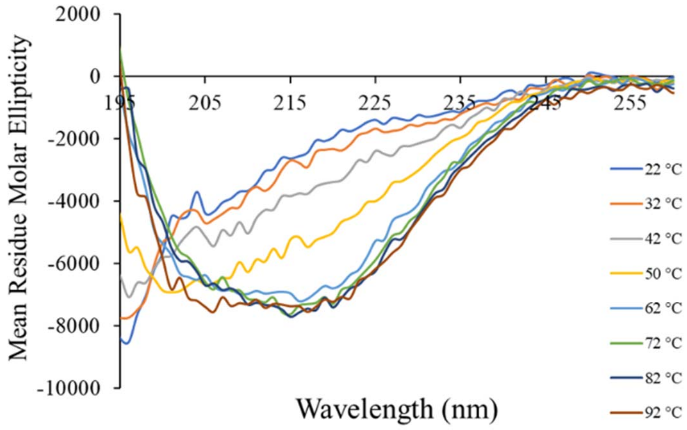

(B)

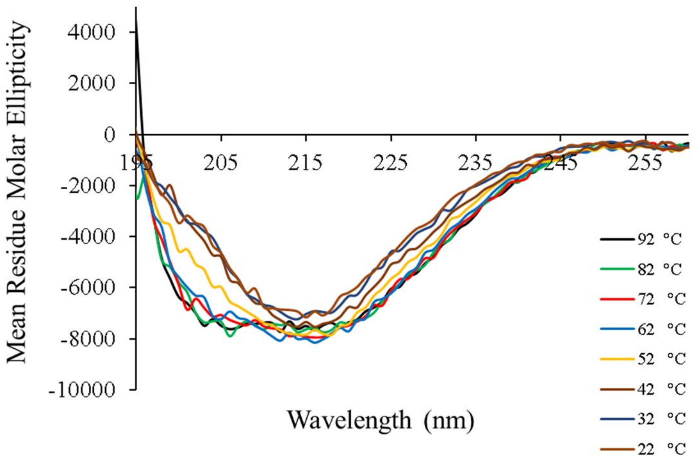

Figure S8 **(A)** CD spectra of MtMce1A$_{36-148}$ from 22 °C to 92 °C. **(B)** CD spectra of MtMce1A$_{36-148}$ from 92 °C to 22 °C.

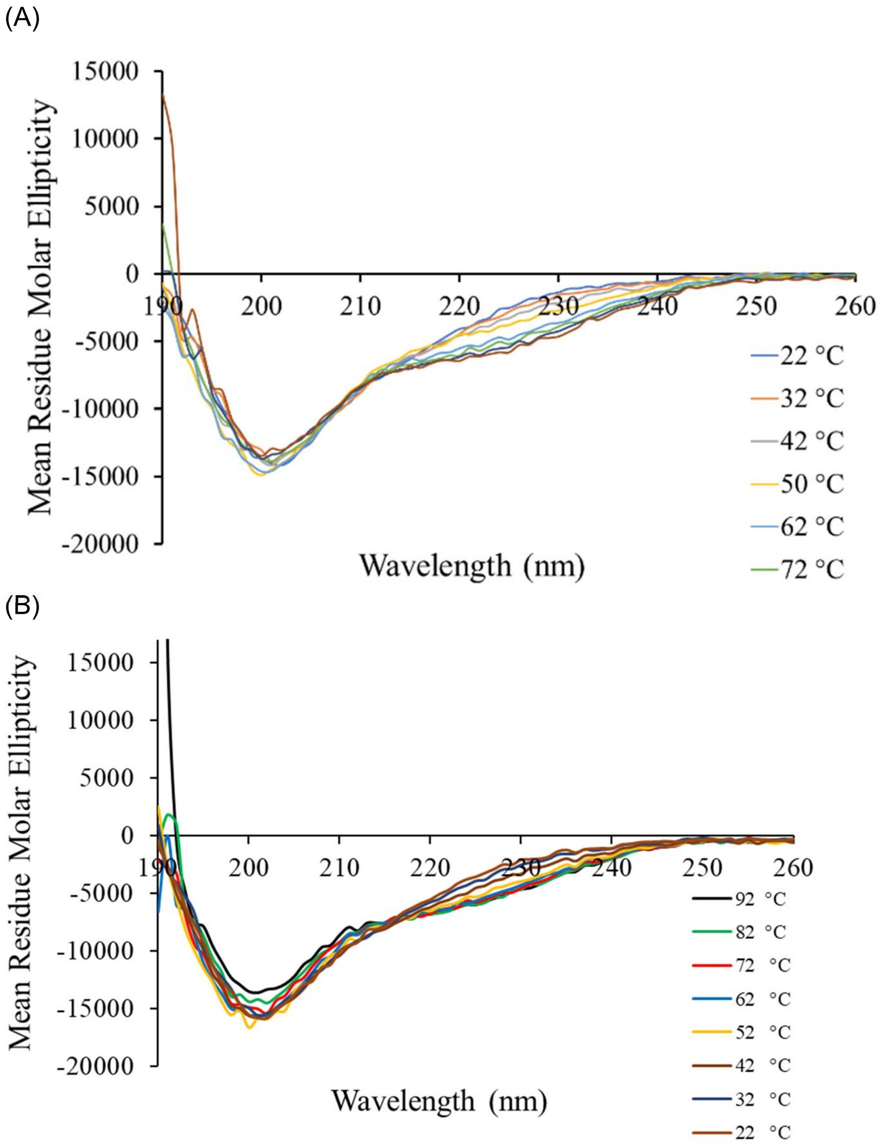

Figure S9 **(A)** CD spectra of MtMce4A$_{39\text{-}140}$ from 22 °C to 72 °C. **(B)** CD spectra of MtMce4A$_{39\text{-}140}$ from 92 °C to 22 °C.

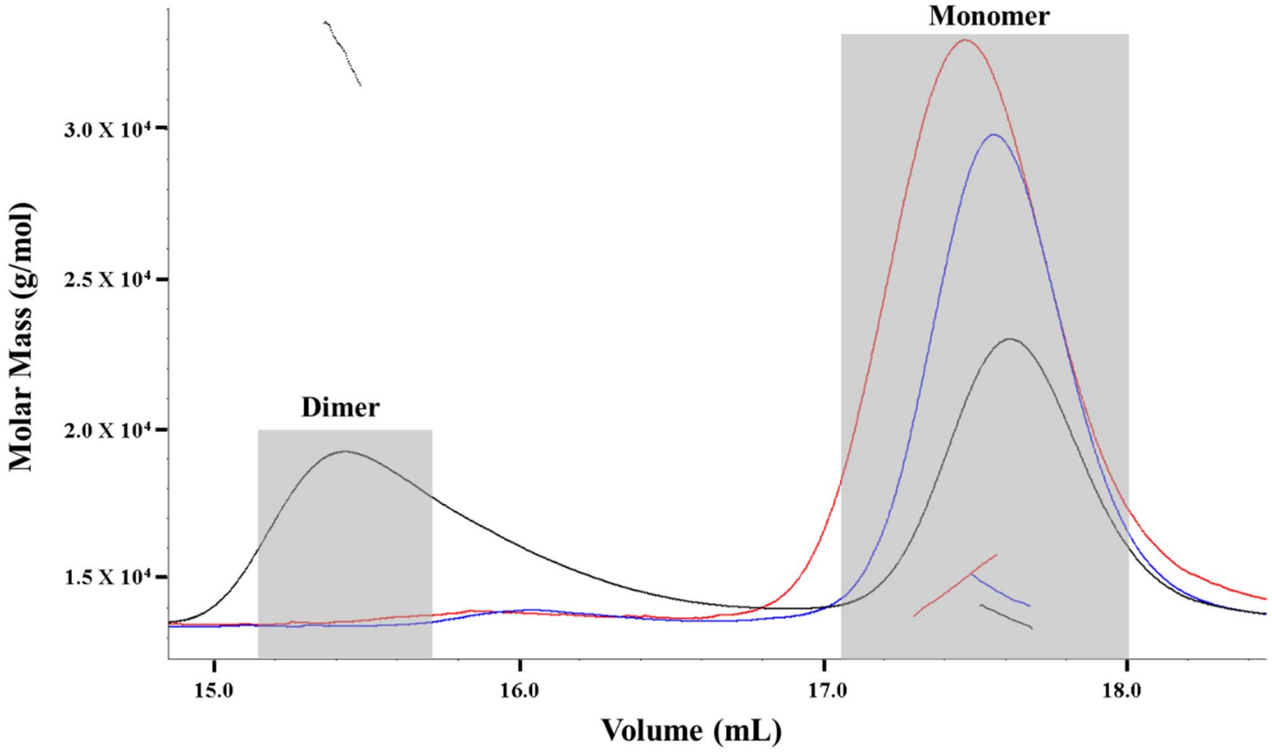

Figure S10 Comparison of SEC-MALS of Mce4A$_{39\text{-}140}$ in three different conditions: Mce4A$_{39\text{-}140}$ in purification buffer (50 mM MOPS, 350 mM NaCl, 10% Glycerol, pH 7.0) in blue, Mce4A$_{39\text{-}140}$ in crystallization buffer (0.1 M MES; 0.7 M ammonium sulfate, pH 6.0) in red, and Mce4A$_{39\text{-}140}$ in crystallization buffer heated up to 50°C in black.

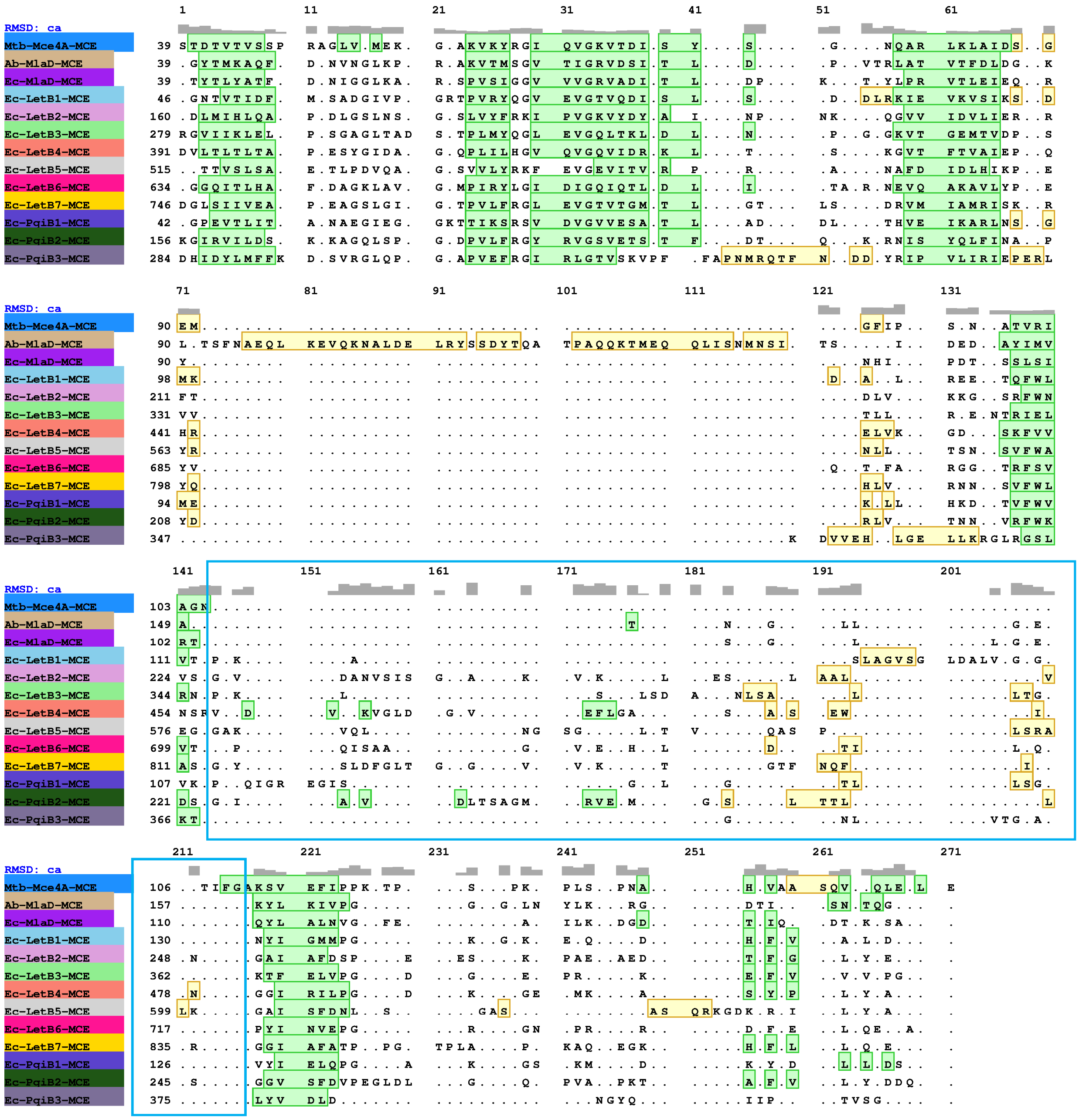

Figure S11 The structure-based sequence aligment of all the known Mce SBP structures (EcMlaD, AbMlaD, EcPqiB1-3, and EcLetB1-7) with the MtMce4A$_{39\text{-}140}$. The alignment was generated using matchmaker (chimera). The β-strands and α-helices are highlighted in green and yellow, respectively. The PLL loop is highlighted in blue box.

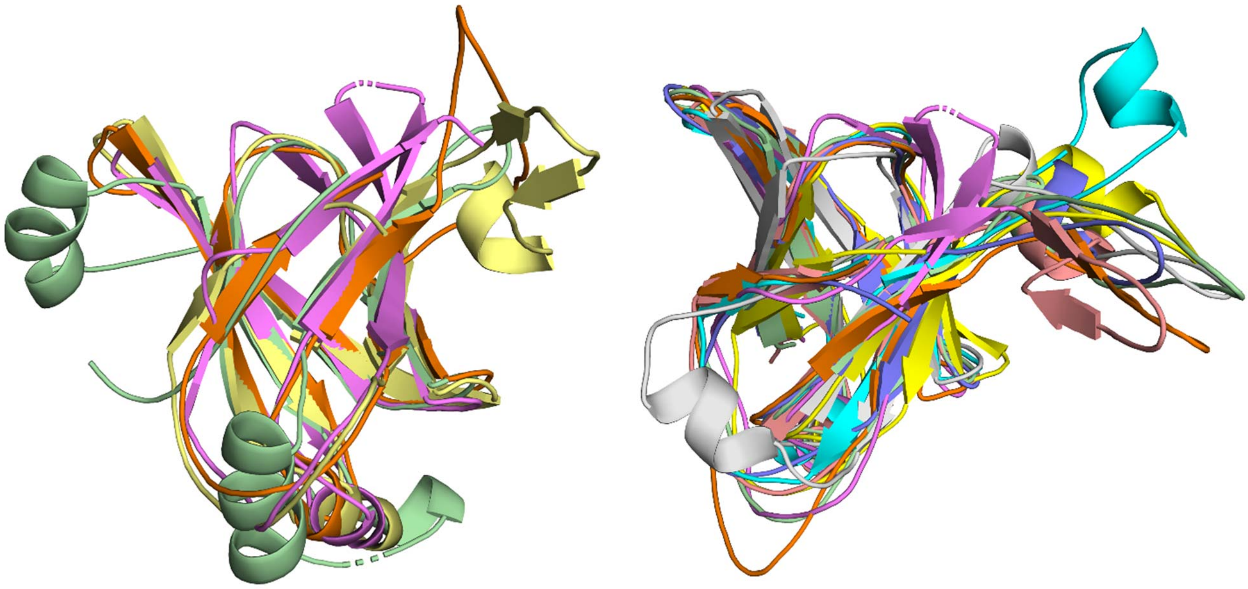

Figure S12 **(A)** Structural superposition of MtMce4A$_{39-140}$ (pink) with the MCE domain of EcPqiB1-3, 5UVN (EcPqiB$_1$; orange, EcPqiB$_2$; yellow, EcPqiB$_3$; green) monomers. **(B)** Structural overlap of MtMce4A$_{39-140}$ (pink) with the MCE domain of EcLetB1-7, 6V0C (EcLetB$_1$; cyan, EcLetB$_2$; green, EcLetB$_3$; yellow, EcLetB$_4$; peach, EcLetB$_5$; gray, EcLetB$_6$; purple, and EcLetB$_7$; orange) monomers.

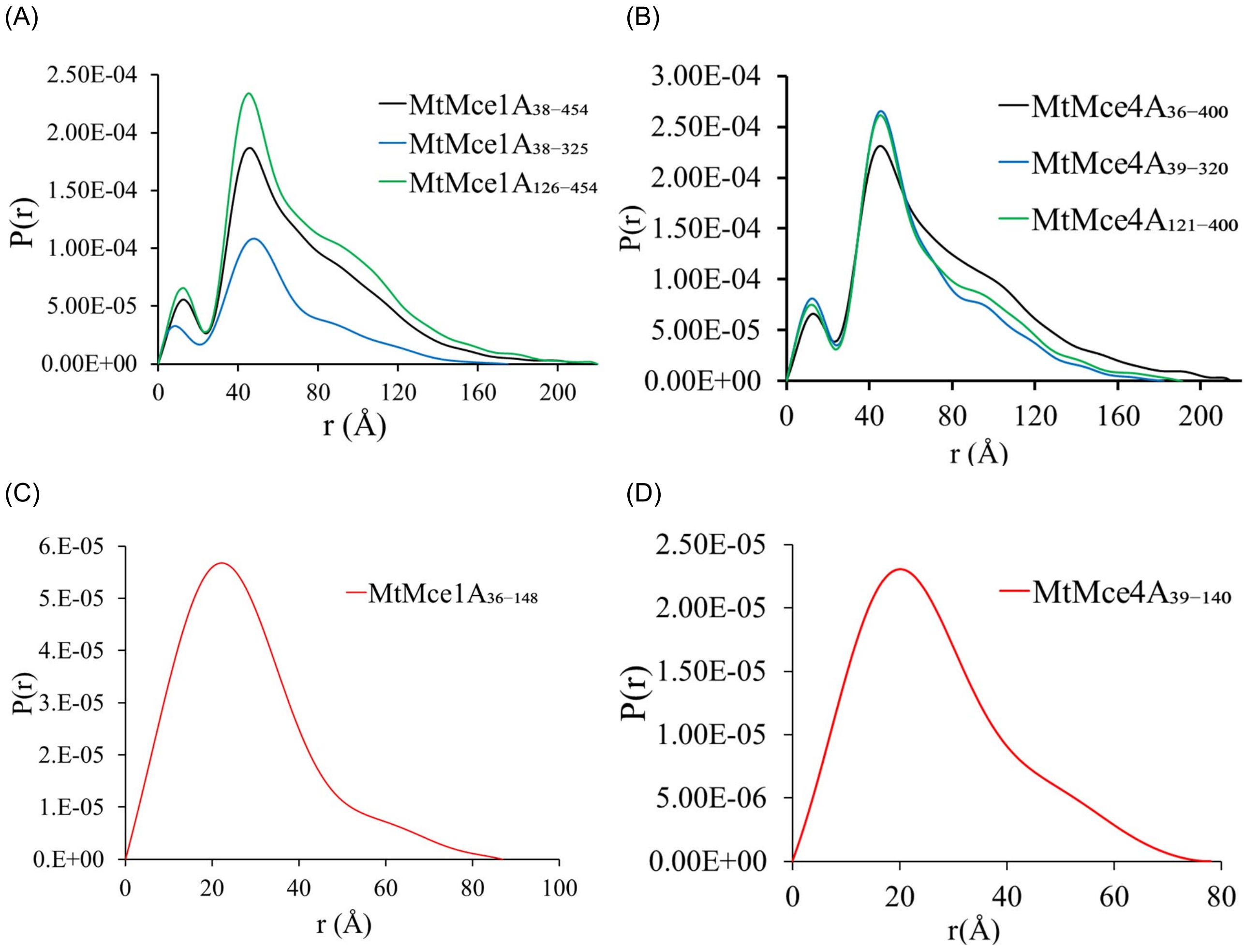

Figure S13 **(A)** The pair distance distribution function, P(r) curves of MtMce1A$_{38\text{-}454}$, MtMce1A$_{38\text{-}325}$ and MtMce1A$_{126\text{-}454}$. **(B)** The P(r) curves of MtMce4A$_{36\text{-}400}$, MtMce4A$_{39\text{-}320}$ and MtMce4A$_{121\text{-}400}$. The P(r) curve of **(C)** MtMce1A$_{36\text{-}148}$ and **(D)** MtMce4A$_{39\text{-}140}$.

(A)

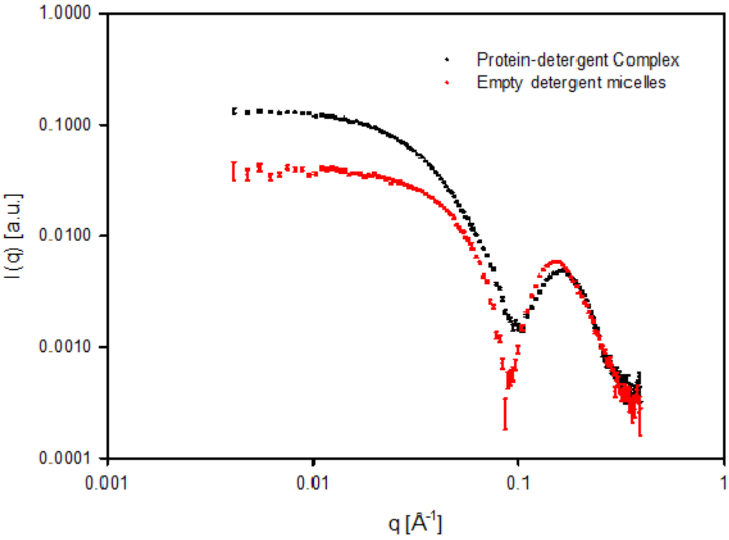

(B)

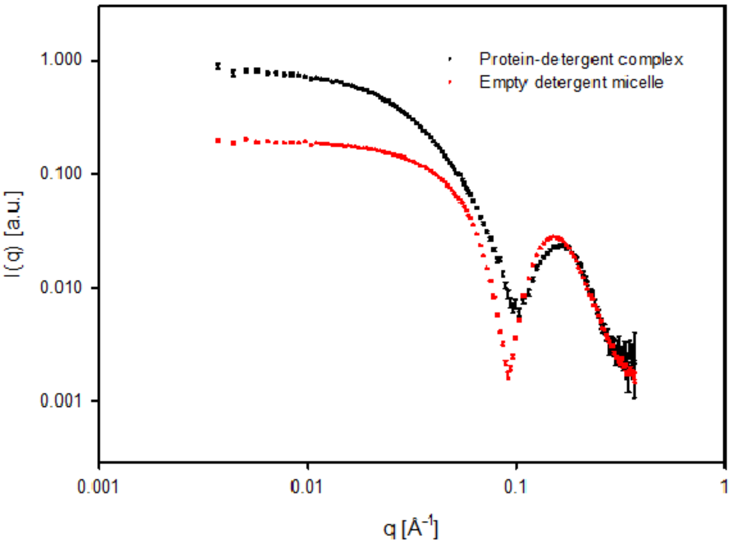

Figure S14 **(A)** SAXS scattering comparison of PDC (black) and empty detergent micelle (red) of MtMce1A38-454. **(B)** SAXS scattering comparison of PDC (black) and empty detergent micelle (red) of MtMce4A36-400.

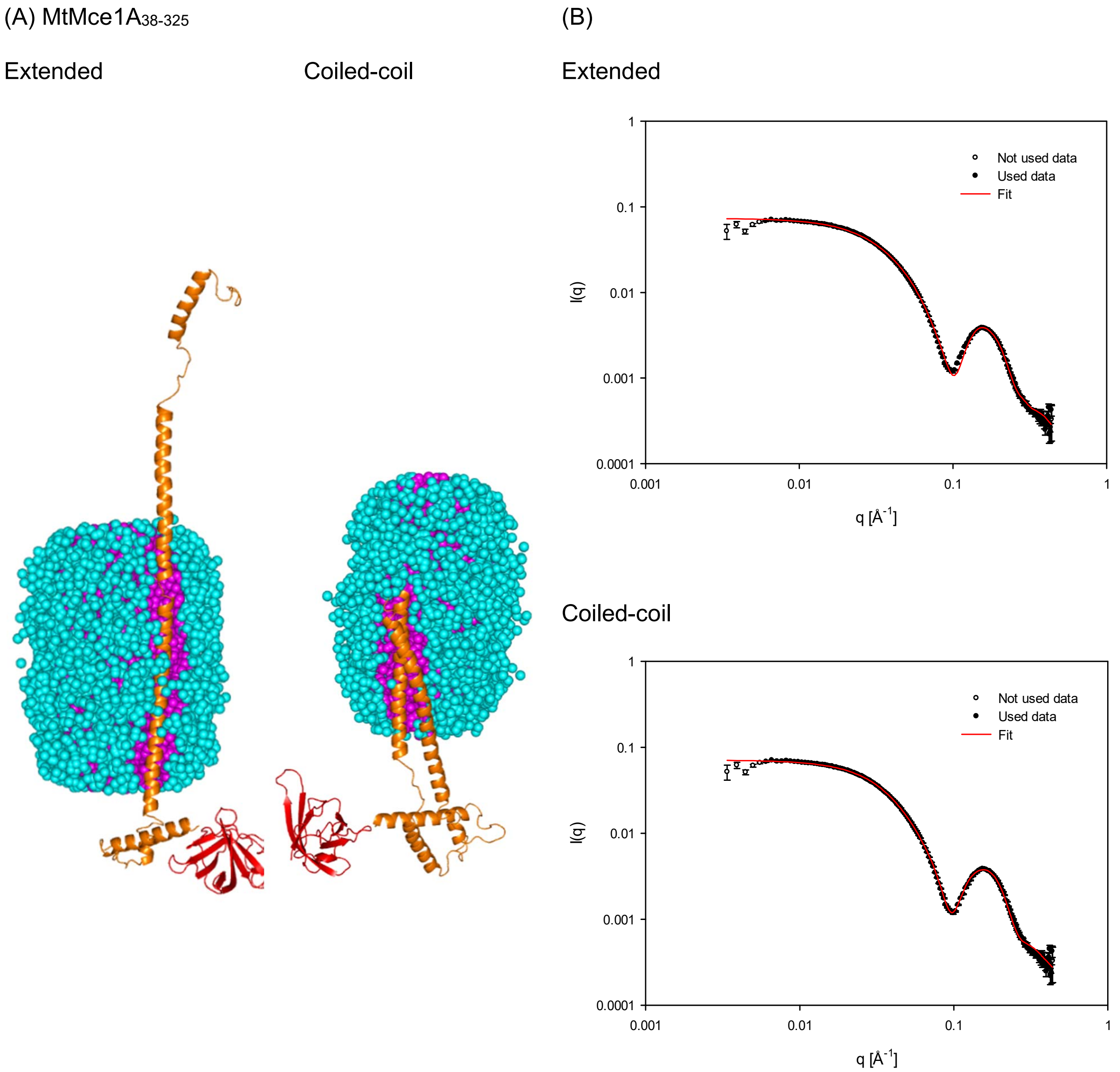

Figure S15 **(A)** The extended (left) and coiled-coil (right) model of MtMce1A$_{38\text{-}325}$. **(B)** The fit of experimental SAXS data with the proposed models of MtMce1A$_{38\text{-}325}$.

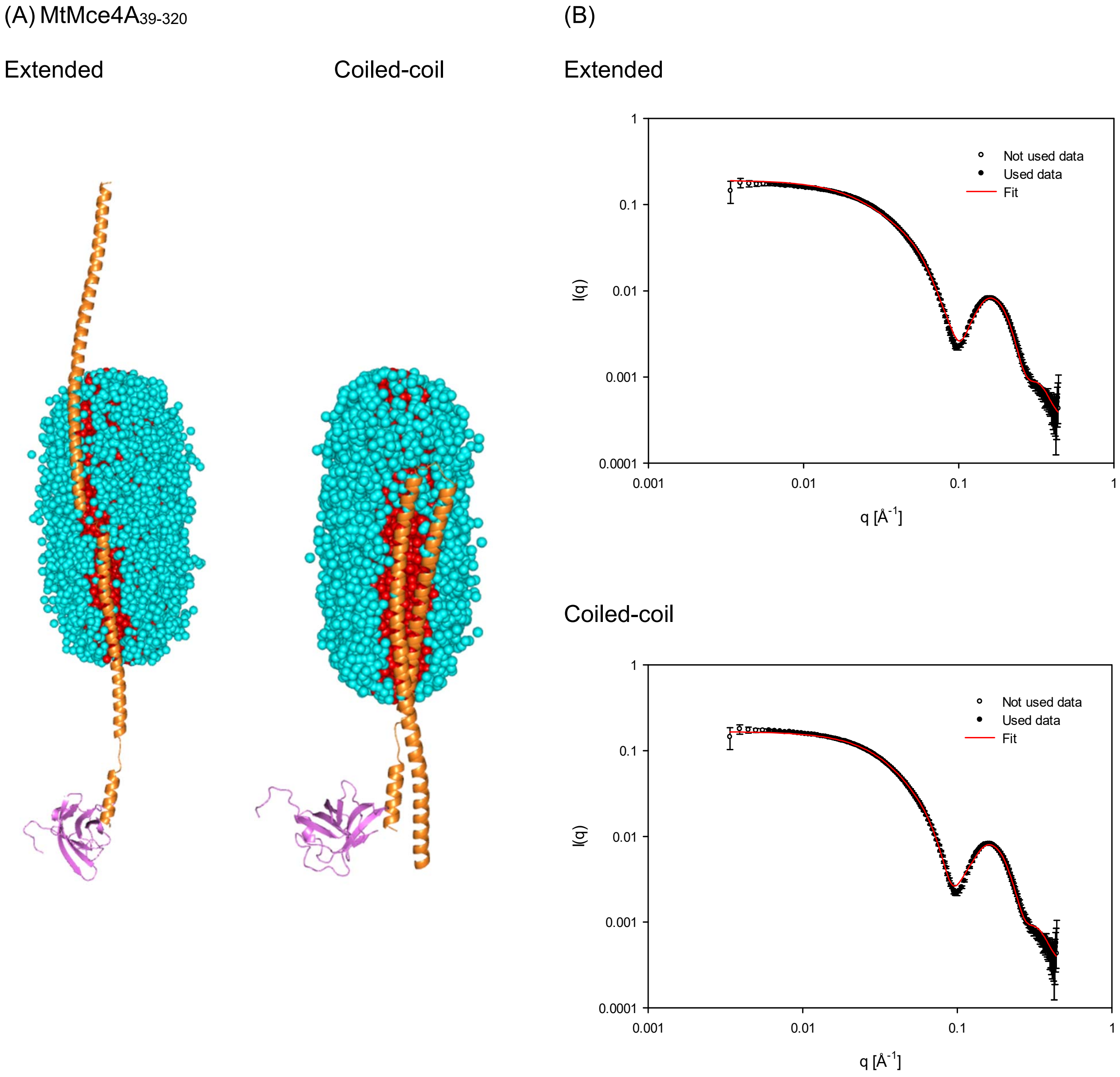

Figure S16 **(A)** The extended (left) and coiled-coil (right) model of MtMce4A$_{39\text{-}320}$. **(B)** The fit of experimental SAXS data with the proposed models of MtMce4A$_{39\text{-}320}$.

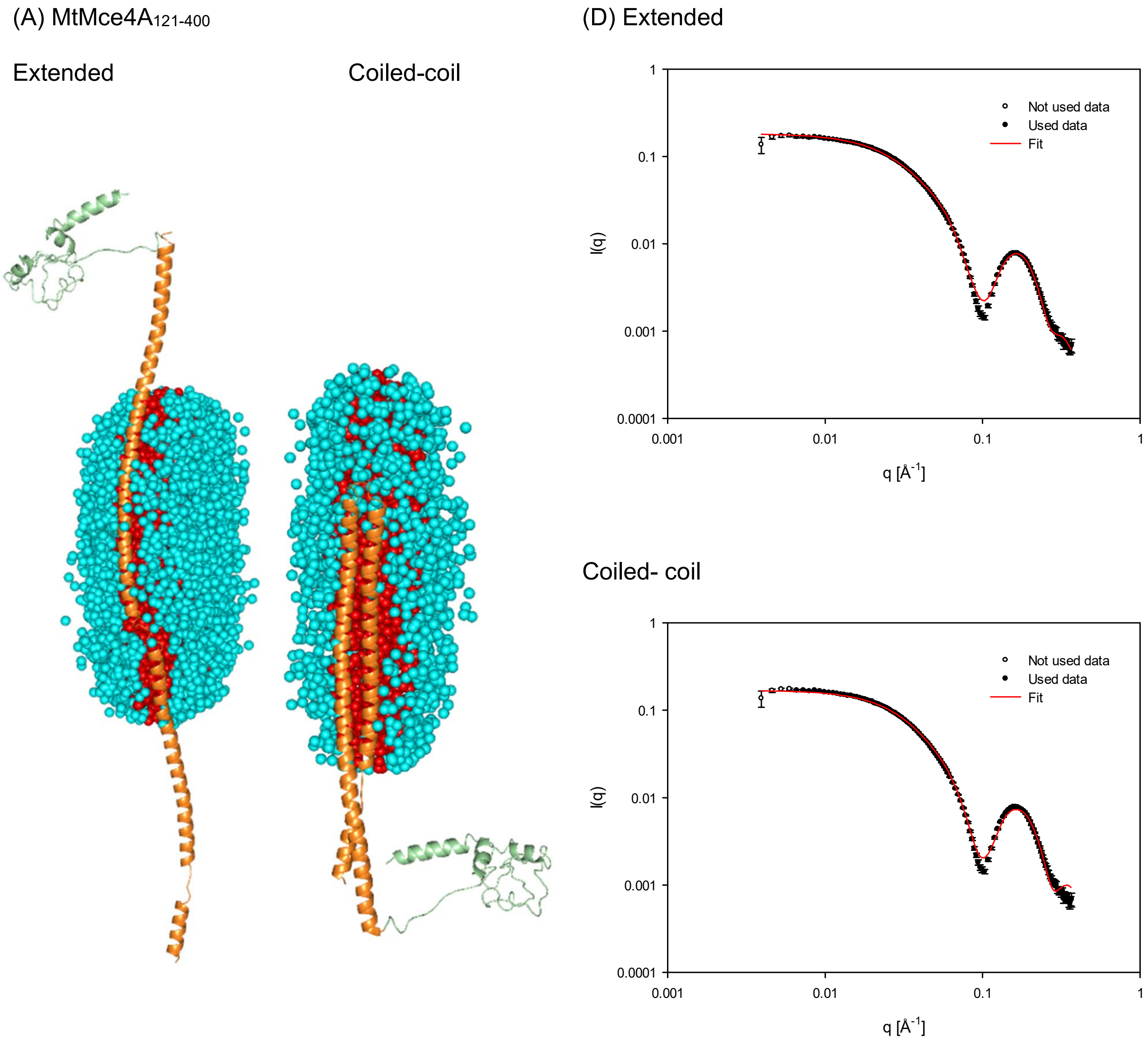

Figure S17 **(A)** The extended and coiled-coil models of MtMce4A$_{121\text{-}400}$. **(B)** The fit of experimental SAXS data with the proposed models of MtMce4A$_{121\text{-}400}$.

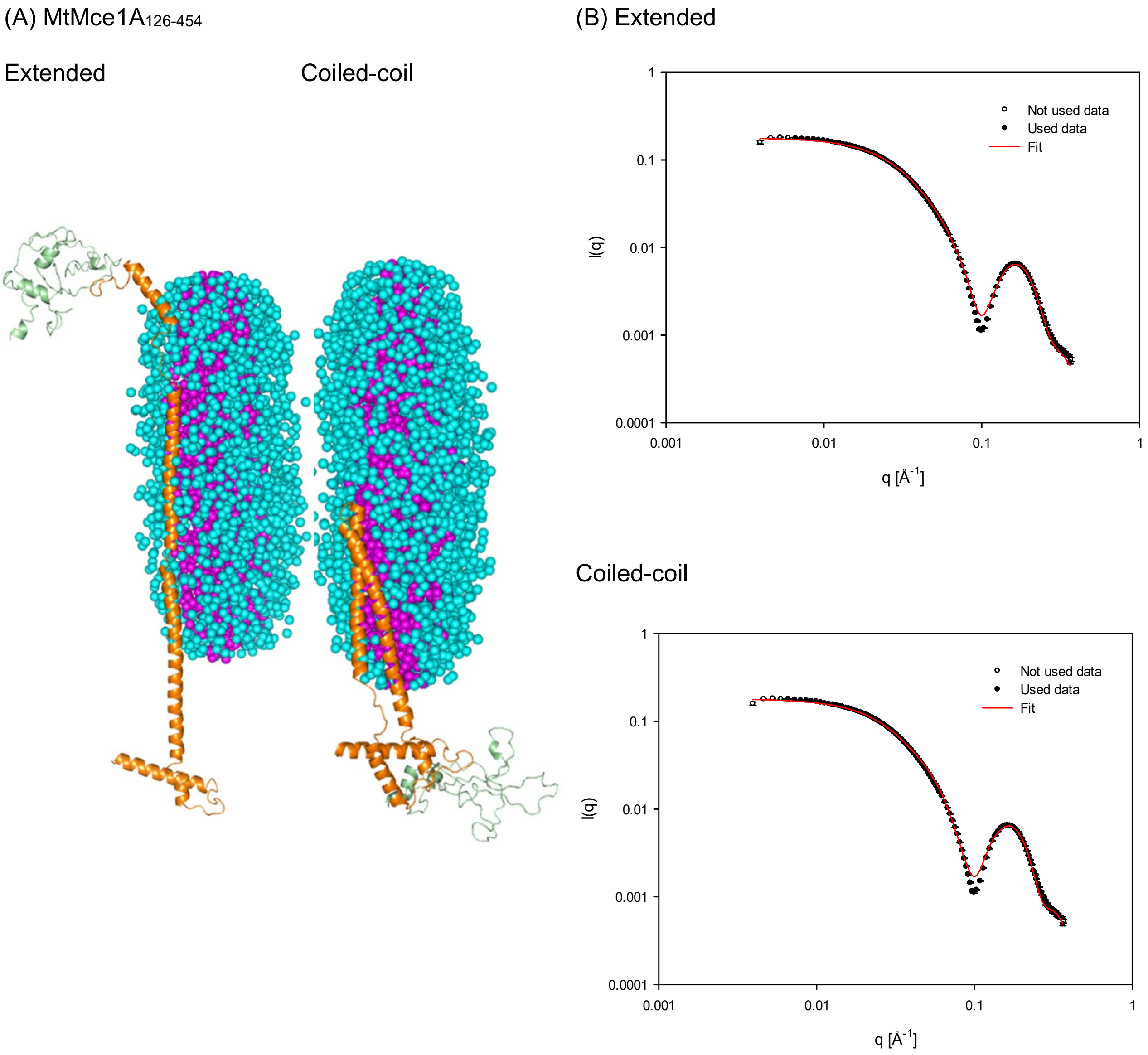

Figure S18 **(A)** The extended and coiled-coil models of MtMce1A$_{126-454}$. **(B)** The fit of experimental SAXS data with the proposed models of MtMce1A$_{126-454}$.

Table S1 List of primers for MtMce1A-1F constructs

Construct Name	Primers
MtMce1A-FP-Nco1	5' TGT ACC ATG GCA ACG ACG CCG GGG AAG CTG AAC 3'
MtMce1A-RP-Xho1	5' CCA GCT CGA GTA ATG GGT TGA TCG TGT TAT CCC CTA CCT G 3'
MtMce1B-FP-Nco1	5' CAC GCC ATG GCA AAA ATC ACT GGA ACC GTC GTC AAA CTC 3'
MtMce1B-RP-Xho1	5' TCC ACT CGA GTA ATT GCG GCG TGC ACC TAC CCG 3'
MtMce1C-FP-Nco1	5' GTG CCC ATG GCA AGA ACG CTG GAA CCA CCC AAC 3'
MtMce1C-RP-Xho1	5' AAG AGA GCT CTA AAT TCT CGC TAC CTC CCG TCA CGC C 3'
MtMce1D-FP-Nco1	5' AGG TCC ATG GCA TTG AGC ACC ATC TTT GAT ATC CGC 3'
MtMce1D-RP-Xho1	5' CAG CAC TCG AGT AAT TGA CCC CCT CCT GCC TCA G 3'
MtMce1E-FP-Nco1	5' AGG ACC ATG GCA ATG AGC GTG CTG GCG CGG AT 3'
MtMce1E-RP-Hind III	5' ATG AAA GCT TTA AGC ACT GGC GAT TTC CCC TTT CTA CCA GCG G 3'
MtMce1F-FP-Nco1	5' CAG CAC TCG AGT AAT TGA CCC CCT CCT GCC TCA G 3'
MtMce1F-RP-Xho1	5' TCG GAA GCT TTA AGC TGG CCG GCG CCA GCA TCT C 3'
MtMce1A$_{38-454}$ FP-Nco1	5' ATA CCC CAT GGC ACG CGG GGA GTT CAC 3'

MtMce1A$_{38-454}$ RP-Xho1	5' CCA GCT CGA GTA ATG GGT TGA TCG TGT TAT CCC CTA CCT G 3'
MtMce1B$_{29-346}$ FP-Nco1	5' GTG ACC ATG GCA CAG ATG CGC TTC GAC CGG AC 3'
MtMce1B$_{29-346}$ RP-Xho1	5' TCC ACT CGA GTA ATT GCG GCG TGC ACC TAC CCG 3'
MtMce1D$_{44-314}$ FP	5'- CTC TCC ATG GCA CTG ACG AAC AAC ACG GTG GTC GCC -3'
MtMce1D$_{44-314}$ RP	5' GGT ACT CGA GGT TAA TGT TCG CCG CCA GCG TC 3'
MtMce1E$_{37-390}$ FP	5'CTACTGAGAATCTTTATTTTCAGGGCGCCATGTCCAATGTGGCGATCCCCGG3'
MtMce1E$_{37-390}$ RP	5' GTGGTGGTGGTGGTGCTCGAGTAAGCACTGGCGATTTCCCC 3'
MtMce1F$_{30-314}$ FP	5' CTG GTC CAT GGC ACG AAT TCC GAG TCT GGT GGG TAT CGG GC 3'
MtMce1F$_{30-314}$ RP	5' GAA TAC TCG AGC GCC GGC GCG AGC GGC -3'
MtMce1A$_{36-148}$ FP	5' CTTTATTTTCAGGGCGCCATGGCACAGTTTCGCGGGGAGTTCACG-3'
MtMce1A$_{36-148}$ RP	5' GTGGTGGTGGTGGTGCTCGAGCTCGGTGGTCACCGACCGTACGTCG-3'
MtMce1A$_{126-454}$ FP	5' CTTTATTTTCAGGGCGCCATGGCA CCGAAAAACCCGACAAAGAGGCGG 3'
MtMce1A$_{126-454}$ RP	5' GTGGTGGTGGTGGTGCTCGAG TAA TGG GTT GAT CGT GTT ATC CCC TAC C 3'
MtMce1A$_{38-325}$ FP	5' CTT TAT TTT CAG GGC GCC ATG GCA CGC GGG GAG TTC ACG CCC AAG3'
MtMce1A$_{38-325}$ RP	5' GTG GTG GTG GTG GTG CTC GAG TGA GTT CGT CCT CAG CGA GTA GCC G 3'

Table S2 List of primers for MtMce4A-4F constructs

Construct Name	Primers
MtMce4A-FP-Nco1	5' CAG ACC ATG GGG TCC GGC GGC GGA TCT CGA C 3'
MtMce4A-RP-Xho1	5' GAG CCT CGA GTA AGA AGT CGT CCC GTT CCG CGA ACG 3'
MtMce4B-FP-Nco1	5' CTT CCC ATG GCG GGC TCG GGC GTT CCC 3'
MtMce4B-RP-Xho1	5' ACT TCT CGA GCA ATT TAG CAA AGG CGC ACC TCC CCT TGC 3'
MtMce4C-FP-Nco1	5' GGA GCC ATG GCA TTG CTA AAT AGG AAG CCA AGT AGC 3'
MtMce4C-RP-Xho1	5' ACC CCT CGA GTA ACG GCG ACT TCG GTC TGA 3'
MtMce4D-FP-Nco1	5' ACC GCC ATG GCA GTG ATG GGC CGG GTC GCC ATG 3'
MtMce4D-RP-Xho1	5' CAG ACT CGA GTA ATC CGC CGC CCC CAT GCT CGC CCG 3'
MtMce4E-FP-Nco1	5' TGG GCC ATG GCA AAC CGA ATC TGG TTG CGC GCC 3'
MtMce4E-RP-Hind III	5' GTC CAA GCT TCA ACT GTC CCG ACG CCG TAC CGG GTG 3'
MtMce4F-FP-Nco1	5' AGG GCC ATG GCA ATG ATC GAC CGA CTC GCC AAG 3'
MtMce4F-RP-Xho1	5' TCT GCT CGA GCA ACA GCT GCC TCG GAT CGC GC 3'
MtMce4A$_{36\text{-}400}$ FP	5' GTG ACC ATG GCA CAG ATG CGC TTC GAC CGG AC 3'
MtMce4A$_{36\text{-}400}$ RP	5' TCC ACT CGA GTA ATT GCG GCG TGC ACC TAC CCG 3'
MtMce4A$_{39\text{-}140}$ FP	5' TTATTTTCAGGGCGCCATGGCCTCTACGGACACCGTCACGGTAT 3’
MtMce4A$_{39\text{-}140}$ RP	5' GTGGTGGTGGTGGTGCTCGAG CTC AAG CTG TAC CTG AGA CGC C 3'
MtMce4A$_{121\text{-}400}$ FP	5' TATTTTCAGGGCGCCATGGCCAAGACGCCGTCGCCCAAGCCG 3'
MtMce4A$_{121\text{-}400}$ RP	5' GTGGTGGTGGTGGTGCTCGAGGAAGTCGTCCCGTTCCGCGAAC 3'
MtMce4A$_{39\text{-}320}$ FP	5’ TTATTTTCAGGGCGCCATGGCCTCTACGGACACCGTCACGGTAT 3’
MtMce4A$_{39\text{-}320}$ RP	5' GTGGTGGTGGTGGTGCTCGAG CAA CAC GAA GCT CGA CGA GGT G 3'
MtMce4A$_{321\text{-}400}$ FP	5’ AGAATCTTTATTTTCAGGGCGCCATGGCC GGT GCG CCG TCG TAC ACC TAT CC 3’
MtMce4A$_{321\text{-}400}$ RP	5'-GTGGTGGTGGTGGTGCTCGAGGAAGTCGTCCCGTTCCGCGAAC 3'

Table S3 List of buffers used in SEC purification

Protein Name	Buffer
MtMce4A, MtMce4C, MtMce4D	50 mM Tris, 500 mM NaCl, 10% Glycerol, 5mM DDM, 1 mM β-ME, pH 8.0
MtMce4B, MtMce4E, MtMce4F	50 mM Tris, 500 mM NaCl, 10% Glycerol, 5 mM FC-12, 1 mM β-ME, pH 8.0
MtMce4A$_{36-400}$, MtMce4A$_{121-400}$,	50 mM Tris, 500 mM NaCl, 10% Glycerol, 5mM DDM, 1 mM β-ME, pH 8.0
MtMce4A$_{39-320}$	50 mM Tris, 500 mM NaCl, 10% Glycerol, 5mM DDM, 1 mM β-ME, pH 8.5
MtMce4A$_{39-140}$	50 mM MOPS, 350 mM NaCl, 10% Glycerol, pH 7.0
MtMce1A$_{36-148}$	50 mM TRIS, 350 mM NaCl, 10% Glycerol, pH 8.5
MtMce1A$_{38-325}$	50 mM Tris, 500 mM NaCl, 10% Glycerol, 5mM DDM, 1 mM β-ME, pH 7.5
MtMce1A$_{126-454}$	50 mM Tris, 350 mM NaCl, 10% Glycerol, 5mM DDM, 1 mM β-ME, pH 7.5
MtMce1A$_{38-454}$	50 mM Tris, 500 mM NaCl, 10% Glycerol, 5mM DDM, 1 mM β-ME, pH 8.0
MtMce1B$_{29-346}$	20 mM Hepes, 500 mM NaCl, 10% Glycerol, 15mM Fos Choline-12, 1 mM β-ME, pH 7.0
MtMce1C, MtMce1D$_{44-314}$	50 mM Tris, 300 mM NaCl, 10% Glycerol, 5mM DDM, 1 mM β-ME, pH 8.0
MtMce1E$_{37-390}$*	50mM Tris, 500mM NaCl, 0.05% C12E9, 1 mM β-ME, pH 8.0
MtMce1F$_{30-314}$	50 mM Tris, 500 mM NaCl, 10% Glycerol, 5mM DDM, 1 mM β-ME, pH 8.0

Lysis buffers for all the purified constructs in DDM and FC-12 had 25 mM of the respective detergents, along with protease inhibitor, lysozyme, DNase and RNase.

*The lysis buffer had 25 mM DDM in this case.

Table S4 SAXS structural parameters for MtMce1A and MtMce4A soluble domains (MtMce1A$_{36-148}$, and MtMce4A$_{39-140}$)

	**MtMce1A$_{36-148}$**	**MtMce4A$_{39-140}$**
Beamline	B21, DLS, UK	B21, DLS, UK
Beam size (μm)	250x250	250x250
Detector	Pilatus 2M	Pilatus 2M
Wavelength (Å)	1.00	1.00
Camera length (m)	4.014	4.014
Protein concentration (mg mL^{-1})	2.0	1.0
Volume injected (μL)	30	30
Buffer used	50mM Tris, 350 mM NaCl, 10% Glycerol, pH 8.5	50mM MOPS, 350 mM NaCl, 10% Glycerol, pH 7.0
Buffer subtraction	SCATTER	SCATTER
Primary analysis	PRIMUS	PRIMUS
Ab-initio shape	DAMMINN	DAMMINN
Atomic structure modelling	Robetta	Crystal structure+ Robetta
3-D representation	PyMOL	PyMOL
**Guinier and P(r) analysis**		
I(0) cm^{-1}	0.023	0.01
R_g (Å)	21.2	20.76
q range (Å)		
qR_g max	1.29	1.29
D_{max} (A)	87	80
χ^2 (total estimate from GNOM)	0.59	0.71
Porod volume, V_p (Å^3)	28759	22236
Molecular mass derived from Q_p, MoW, V_c, size & shape (kDa)	11.13, 11.60, 15.44, 19.9	10.03, 10,30, 14.22, 16.37
**DAMMIN parameters**		
Symmetry, anisotropy assumption	P1 unknown	P1 unknown

χ^2	1.26	1.104
**Atomistic modelling**		
Model used	Elongated and compact	Elongated and compact
**Fit**		
χ^2	4.2, 14.0	2.0, 10.0
**SASBDB accession codes**	SASDJU9	SASDJV9

Table S5 SAXS structural parameters for MtMce1A domains (MtMce1A$_{38-454}$, MtMce1A$_{126-454}$, and MtMce1A$_{38-325}$)

	**MtMce1A$_{38-454}$**	**MtMce1A$_{126-454}$**	**MtMce1A$_{38-325}$**
Beamline	B21, DLS, UK	B21, DLS, UK	B21, DLS, UK
Beam size (μm)	250x250	250x250	250x250
Detector	Pilatus 2M	Pilatus 2M	Pilatus 2M
Wavelength (Å)	1.00	1.00	1.00
Camera length (m)	4.014	4.014	4.014
SEC-SAXS column	Superdex 200 increase 3.2/300	Superdex 200 increase 3.2/300	Superdex 200 increase 3.2/300
Protein concentration (mg mL^{-1})	5	5.2	6.0
Volume injected (μL)	55	55	55
Flow rate (mL min^{-1})	0.075	0.15	0.06
Buffer used	50 mM Tris, 500 mM NaCl, 10% Glycerol, 5mM DDM, 1 mM β-ME, pH 8.0	50mM Tris, 350 mM Nacl, 10%glycerol, 5 mM DDM, 1mM β -ME, pH 7.5	50mM Tris, 500mM NaCl, 10% Glycerol, 5mM DDM, 1mM β -ME, pH 7.5
Buffer subtraction	SCATTER	SCATTER	SCATTER
Primary analysis	PRIMUS	PRIMUS	PRIMUS
Ab-initio shape	-	-	-
Atomic structure modelling	Swiss-model, iTasser	Swiss-model, iTasser	Swiss-model, iTasser
3-D representation	PyMOL	PyMOL	PyMOL
**Guinier and P(r) analysis**			
I(0) cm^{-1}	0.15	0.18	0.07
R_g (Å)	52.8	54.86	45.82
qR_g max	1.29	1.29	1.29
D_{max} (Å)	216	220	175
χ^2 (total estimate from GNOM)	0.63	0.62	0.67

**R factor analysis**			
R factor $= \frac{\sum \|I(q_i) - I_{model}(q_i)\|}{\sum I(q_i)}$ (Extended model, coiled-coil)	1.2 %, 1.3 %	1.5 %, 2.2 %	1.0 %, 0.9 %
Weighted R factor= $\left(\frac{\sum (I(q_i)-I_{model}(q_i))^2/\sigma(I(q_i))^2}{\sum I(q_i)^2/\sigma(I(q_i))^2}\right)^{0.5}$ (Extended model, coiled-coil)	1.9 %, 2.4 %	2.3 %, 3.3 %	1.9 %, 1.6 %
**SASBDB accession codes**	SASDK32	SASDK22	SASDJZ9

Table S6 SAXS structural parameters for MtMce4A domains (MtMce4A$_{36\text{-}400}$, MtMce4A$_{121\text{-}400}$, and MtMce4A$_{39\text{-}320}$)

	**MtMce4A$_{36\text{-}400}$**	**MtMce4A$_{121\text{-}400}$**	**MtMce4A$_{39\text{-}320}$**
Beamline	B21, DLS, UK	B21, DLS, UK	B21, DLS, UK
Beam size (µm)	250x250	250x250	250x250
Detector	Pilatus 2M	Pilatus 2M	Pilatus 2M
Wavelength (Å)	1.00	1.00	1.00
Camera length (m)	4.014	4.014	4.014
SEC-SAXS column	Superdex 200 increase 3.2/300	Superdex 200 increase 3.2/300	Superdex 200 increase 3.2/300
Protein concentration (mg mL^{-1})	5	5	5
Volume injected (µL)	55	55	55
Flow rate (mL min^{-1})	0.075	0.15	0.06
Buffer used	50 mM Tris, 500 mM NaCl, 10% Glycerol, 5mM DDM, 1 mM β-ME, pH 8.0	50 mM Tris, 500 mM NaCl, 10% Glycerol, 5mM DDM, 1 mM β-ME, pH 8.0	50 mM Tris, 500 mM NaCl, 10% Glycerol, 5mM DDM, 1 mM β-ME, pH 8.5
Buffer subtraction	SCATTER	SCATTER	SCATTER
Primary analysis	PRIMUS	PRIMUS	PRIMUS
Ab-initio shape	-	-	-
Atomic structure modelling	Mce4A$_{39\text{-}140}$ Crystal structure, iTasser	Mce4A$_{39\text{-}140}$ Crystal structure, iTasser	Mce4A$_{39\text{-}140}$ Crystal structure, iTasser
3-D representation	PyMOL	PyMOL	PyMOL
**Guinier and P(r) analysis**			
I(0) cm^{-1}	0.20	0.17	0.17
R_g (Å)	57.35	50.26	47.54
qR_g max	1.29	1.29	1.29
D_{max} (Å)	215	190.88	181.90
χ^2 (total estimate from GNOM)	0.51	0.68	0.69

**R factor analysis**			
R factor $= \frac{\sum \vert I(q_i) - I_{model}(q_i)\vert}{\sum I(q_i)}$ (Extended model, coiled-coil)	1.6 %, 2.5 %	2.0 %, 2.3 %	4.3 %, 1.8 %
Weighted R factor $\left(\frac{\sum (I(q_i)-I_{model}(q_i))^2/\sigma(I(q_i))^2}{\sum I(q_i)^2/\sigma(I(q_i))^2}\right)^{0.5}$ (Extended model, coiled-coil)	3.6 %, 3.4 %	5.4 %, 4.2 %	3.6 %, 2.3 %
**SASBDB accession codes**	SASDJW9	SASDJX9	SASDJY9

Table S7 Percent identity matrix for MtMce1A-1F created by Clustal Omega

**MtMce1A-1F**						
	MtMce1A	MtMce1B	MtMce1C	MtMce1D	MtMce1E	MtMce1F
MtMce1A	100.00	17.07	18.11	20.15	16.76	18.88
MtMce1B	17.07	100.00	21.52	18.64	17.77	16.13
MtMce1C	18.11	21.52	100.00	20.67	20.68	20.31
MtMce1D	20.15	18.64	20.67	100.00	17.63	18.41
MtMce1E	16.76	17.77	20.68	17.63	100.00	18.32
MtMce1F	18.88	16.13	20.31	18.41	18.32	100.00

Table S8 Percent identity matrix for MtMce4A-4F created by Clustal Omega

**MtMce4A-4F**						
	MtMce4A	MtMce4B	MtMce4C	MtMce4D	MtMce4E	MtMce4F
MtMce4A	100.00	17.27	15.36	19.27	18.69	21.43
MtMce4B	17.27	100.00	19.55	22.65	16.16	17.54
MtMce4C	15.36	19.55	100.00	28.39	19.86	16.38
MtMce4D	19.27	22.65	28.39	100.00	17.03	20.11
MtMce4E	18.69	16.16	19.86	17.03	100.00	24.14
MtMce4F	21.43	17.54	16.38	20.11	24.14	100.00

Table S9 Secondary structure content of MtMce1A and MtMce4A domains

	**Helix**	**Anti-Parallel β-strand**	**Parallel β-strand**	**β-turn**	**Random coil**
$MtMce1A_{36\text{-}148}$	2.3%	40.6%	3.7%	13.5%	39.9%
$MtMce1A_{38\text{-}325}$	27.9%	13.3%	9.8%	18.0%	33.7%
$MtMce1A_{126\text{-}454}$	30.6%	11.7%	8.8%	17.6%	30.6%
$MtMce1A_{38\text{-}454}$	38.3%	8.5%	7.2%	16.3%	25.7%
$MtMce4A_{39\text{-}140}$	6.7%	24.2%	3.9%	13.2%	52%
$MtMce4A_{39\text{-}320}$	28.0%	12.8%	9.9%	17.9%	34.8%
$MtMce4A_{121\text{-}400}$	33.8%	10.5%	7.8%	17.1%	26.8%
$MtMce4A_{36\text{-}400}$	37.5%	8.3%	7.1%	16.3%	25.6%

Table S10 Secondary structure comparison of $MtMce4A_{39\text{-}140}$ in solution and crystal structure

	In solution	Crystal structure
alpha-Helix (distorted)	6.70	4.10
β-strands (parallel and Anti-parallel)	28.10	39.04
β-turn	13.20	8.90
Others	52.00	47.95
